# Filler-Geometry-Dependent Crystallinity, Melt Flow, and Mechanical Response of Glass-Filled PHBV + PBAT + TPS Composites

**DOI:** 10.3390/polym18182233

**Published:** 2026-09-13

**Authors:** Magdalena Pantoł, Klaudia Porzezinska, Krzysztof Nowik, Ewa Borucinska-Parfieniuk, Mehmet Aladag, Adrian Dubicki, Krzysztof J. Kurzydłowski, Izabela B. Zgłobicka

**Affiliations:** 1 Faculty of Mechanical Engineering, Bialystok University of Technology, Wiejska 45C, 15-351 Bialystok, Poland; magdalena.pantol@sd.pb.edu.pl or magdalena.pantol@grupaazoty.com (M.P.); klaudiaporzezinskaa@gmail.com (K.P.); krzysztof.nowik@sd.pb.edu.pl (K.N.); ewa.borucinska-parfieniuk@sd.pb.edu.pl (E.B.-P.); mehmet.aladag@sd.pb.edu.pl (M.A.); a.dubicki@pb.edu.pl (A.D.); k.kurzydlowski@pb.edu.pl (K.J.K.); 2 Grupa Azoty S.A., Kwiatkowskiego 8, 33-100 Tarnów, Poland

**Keywords:** poly(3-hydroxybutyrate-co-3-hydroxyvalerate) (PHBV), poly(butylene adipate-co-terephthalate) (PBAT), thermoplastic starch (TPS), glass reinforcement, properties

## Abstract

The structural, processing, and mechanical response of a multiphase poly(3-hydroxybutyrate-co-3-hydroxyvalerate) (PHBV)/poly(butylene adipate-co-terephthalate) (PBAT)/thermoplastic starch (TPS) matrix to two distinct glass fillers was investigated. Glass fibers and hollow glass spheres were incorporated by melt compounding and injection molding, while the unfilled blend served as the reference. Differential scanning calorimetry, X-ray diffraction, melt-flow-rate measurements, helium pycnometry, scanning electron microscopy with deep-learning-based segmentation, tensile, and Charpy impact tests were applied. At higher filler contents, the composite-level XRD-based crystallinity index decreased to approximately 47%, whereas the Scherrer-derived PHBV (110) coherent-domain size remained within approximately 21–24 nm. Preferred orientation, assessed independently from the PHBV reflection-intensity ratio, varied with filler type and content. Glass fibers progressively reduced melt flow and were associated with an increase in tensile modulus from 2.06 to 3.45 GPa and maximum tensile stress from 23.46 to 27.32 MPa at the highest investigated fiber content. Hollow glass spheres produced a non-monotonic melt-flow response, while the reduction in tensile performance at higher contents coincided with decreasing interparticle spacing and increasing specific external polymer–glass interfacial area. Within the analyzed SEM fields, no pronounced filler-rich clustering was evident. Notched specimens remained brittle, whereas unnotched specimens retained impact strength above 10 kJ × m^−2^. Overall, the two filler geometries exhibited distinct relationships among apparent melt flowability, crystalline organization, quantitative microstructural descriptors, and mechanical response.

## 1. Introduction

Biodegradable polymer matrices are increasingly investigated as potential alternatives to conventional petroleum-based plastics, particularly for products requiring a limited-service life or reduced environmental persistence. Among biodegradable polyesters, poly(3-hydroxybutyrate-co-3-hydroxyvalerate) (PHBV) is particularly attractive because it is bio-based, biodegradable, and can be produced from renewable or residual biomass streams. Its relatively high stiffness and crystallinity are advantageous for maintaining dimensional stability. However, neat PHBV is limited by its brittleness, relatively high cost, and narrow processing window, which restrict its broader engineering use. Blending PHBV with more ductile or lower-cost biodegradable polymers is therefore commonly applied to improve its processability and balance its mechanical performance.

In the present study, PHBV was combined with poly(butylene adipate-co-terephthalate) (PBAT) and thermoplastic starch (TPS) to obtain a polymer matrix while balancing stiffness, processability, and renewable material content. PHBV is a semicrystalline aliphatic polyester whose relatively regular chain structure promotes crystallization and provides stiffness, but also contributes to brittleness and limited deformation capacity. PBAT contains flexible aliphatic adipate segments and aromatic terephthalate units and was introduced as a more ductile biodegradable copolyester that is capable of improving melt processability and reducing the brittleness of PHBV-rich blends [1,2,3]. TPS, composed mainly of plasticized amylose and amylopectin chains rich in hydroxyl groups, was incorporated as a renewable and comparatively low-cost component contributing a polar, predominantly amorphous biodegradable phase.

Because PHBV and PBAT differ markedly from TPS in polarity, molecular architecture, and crystallization behavior, the PHBV + PBAT + TPS system should be considered a multiphase physical blend rather than a fully miscible molecular system [3,4]. Its properties are consequently controlled not only by the intrinsic properties and proportions of the three constituents, but also by phase continuity, polymer–polymer interphases, crystalline organization, and processing-induced morphology. The crystalline contribution is expected to be dominated by PHBV, with a smaller contribution from PBAT and a predominantly amorphous TPS-rich fraction. Changes in the organization of these phases may therefore affect stiffness, deformation, and fracture even when the nominal matrix composition remains unchanged. Moreover, the flow behavior of starch-containing biodegradable polyester blends is influenced by the rheological contrast between the constituent phases and their phase morphology [5].

The introduction of rigid inorganic fillers adds another level of structural complexity to such a multiphase polymer matrix. Besides their direct contribution to load transfer, inorganic surfaces may modify heterogeneous nucleation, crystal growth, and the overall crystalline organization of PHBV. The direction and magnitude of these changes depend on filler chemistry, concentration, dispersion, and processing history [6,7]. Filler geometry may also alter the local flow field during melt processing. High-aspect-ratio fibers undergo shear-induced orientation and may simultaneously influence the molecular orientation of the surrounding polymer matrix [8]. In particle-filled melts, the flow response is additionally governed by particle size, volume fraction, and the development of interparticle interactions as the distance between neighboring particles decreases [9]. Consequently, the response of a filled multiphase blend cannot be predicted solely from the intrinsic stiffness or nominal content of the filler, but must also be considered in terms of matrix crystallization, melt flowability, processing-induced orientation, and polymer–filler interfacial constraints.

Glass fibers and glass spheres represent two fundamentally different reinforcement geometries. Owing to their high aspect ratio, glass fibers can transfer load along their longitudinal direction and may enhance stiffness through interfacial shear and flow-induced orientation. Their reinforcing efficiency depends on fiber content, diameter, aspect ratio, orientation, dispersion, and fiber–matrix adhesion [10,11,12,13]. At excessive contents, however, increased flow resistance, fiber–fiber interactions, incomplete wetting, or processing-related defects may limit further improvement and promote premature failure.

Glass spheres do not provide the same long-range anisotropic load-transfer mechanism. At low or moderate contents, they may behave as isolated rigid inclusions and modify local stress distribution without strongly disrupting matrix continuity [14,15,16]. At higher contents, decreasing interparticle distances and increasing polymer–glass interfacial area may promote stress concentration, particle–matrix debonding, and micro-void formation, particularly when interfacial adhesion is insufficient [17]. The effect of spherical fillers may also depend strongly on loading mode. Under dynamic or impact loading, interfacial debonding, microcracking, crack-path deflection, and localized deformation of the surrounding matrix may contribute to energy dissipation, even when the same particles reduce strength under quasi-static tensile loading [18,19,20,21]. Thus, spherical fillers should not be treated simply as stiffness-enhancing additives, because their contribution may change with filler content, interparticle spacing, interfacial area, and loading conditions.

Previous studies have addressed individual elements of this problem, but generally in separate material systems or with a narrower set of descriptors. Vidakis et al. [22], for example, compared glass beads, flakes, and powders in polylactic-acid-based composites and demonstrated a strong dependence of mechanical behavior on filler morphology and content. Other studies have shown that inorganic fillers can modify crystallization and processing behavior in PHBV- and PHBV/PBAT-based systems [6,7], whereas Laufer et al. [9] demonstrated the importance of particle concentration and interparticle interactions for the flow behavior of glass-bead-filled polymer melts. These studies establish the relevance of filler morphology, crystallization, and particle interactions individually, but they do not resolve whether geometrically distinct fillers produce coordinated or divergent structural, processing, and mechanical responses when introduced into the same multiphase polymer matrix. In particular, an integrated comparison combining composite-level crystalline organization, apparent melt flowability, quantitative filler spacing, specific external polymer–glass interfacial area, and loading-mode-dependent mechanical behavior has not been reported for an identically formulated and processed PHBV + PBAT + TPS matrix.

Accordingly, the objective of this study was to determine how glass-filler geometry modifies the structural, processing, and mechanical response of an identically formulated PHBV + PBAT + TPS matrix. Glass fibers and glass spheres were introduced at several volume fractions by melt compounding followed by injection molding, while an identically processed unfilled blend served as the reference. Differential scanning calorimetry and X-ray diffraction were used to examine thermal transitions, apparent crystallinity, crystalline-domain size, and processing-induced texture. Melt flow rate measurements were used to assess relative melt flowability, whereas helium pycnometry and scanning electron microscopy combined with deep-learning-based segmentation provided information on density, filler distribution, inter-object spacing, and specific polymer–glass interfacial area. These results were related to tensile and Charpy impact behavior.

The central scientific question was therefore not whether filler geometry affects composite behavior per se, but whether two fundamentally different glass-filler geometries produce parallel or divergent structural, processing, and mechanical responses when matrix composition and processing route are held constant. The novelty of this study lies in identifying, within this controlled PHBV + PBAT + TPS system, a partial decoupling between composite-level crystalline descriptors, apparent melt flowability, quantitative filler arrangement, and mechanical response, demonstrating that none of these descriptors alone is sufficient to characterize the geometry-dependent composite response. From an application perspective, the investigated materials are intended primarily as candidates for low-loaded, non-safety-critical injection-molded components in which moderate stiffness, dimensional stability, processability, and acceptable impact resistance are required rather than high structural strength. Potential applications may include protective covers, housings, spacers, support elements, and other lightly loaded technical components. These examples represent prospective application areas rather than validated end uses, as application-specific durability and service-life testing were beyond the scope of the present study.

## 2. Materials and Methods

### 2.1. Materials

The polymer matrix consisted of 49.75 wt.% poly(3-hydroxybutyrate-co-3-hydroxyvalerate) (PHBV), 24.875 wt.% poly(butylene adipate-co-terephthalate) (PBAT), 24.875 wt.% thermoplastic starch (TPS), and 0.50 wt.% calcium stearate. PHBV was used as the main semicrystalline biodegradable polyester component; PBAT as a flexible biodegradable copolyester intended to improve processability and reduce brittleness; and TPS as a renewable, low-cost biodegradable component. Accordingly, biodegradability in the present study refers to the polymer-matrix constituents and not to the complete glass-filled composite.

PHBV was a commercially available grade ENMAT Y1000P supplied by Kanghui (Kanghui New Material Technology Co., Ltd., Liaoning Province, China). PBAT was a commercially available grade TH801T supplied by Tunhe, China (Xinjiang Blue Ridge Tunhe Sci.&Tech. Co., Ltd., Changji City, Xinjiang, China). Thermoplastic starch envifill^®^ TPS was supplied by Grupa Azoty, Tarnów, Poland. The TPS grade was based on potato starch and additives of natural origin and was intended for processing by extrusion and blow molding, either as a base material or as an additive for biodegradable polymer blends. The selected properties of the polymer components are summarized in Table 1. The values were taken from the corresponding technical data sheets.

Calcium stearate (FACI Calcium Stearate S, FACI/Omya) was used as a processing aid at 0.50 wt.% of the polymer-matrix formulation. A reference material density of 1.12 g × cm^−3^ was used for calcium stearate [23].

Chopped glass fibers (ChopVantage HP 3730, Nippon Electric Glass, Otsu, Shiga, Japan) and hollow glass spheres (product 440345, Supelco/Sigma-Aldrich, Darmstadt, Germany) were used as inorganic reinforcements. According to the supplier specification, the hollow glass spheres have a nominal external particle size of 9–13 µm and a reported density of 1.10 g × cm^−3^. In the present calculations, this value was treated as the effective density of the complete hollow particles. Their selected properties are summarized in Table 2. The quantitatively determined particle-size distribution observed by SEM is discussed in Section 3.4.

**Figure 1:**
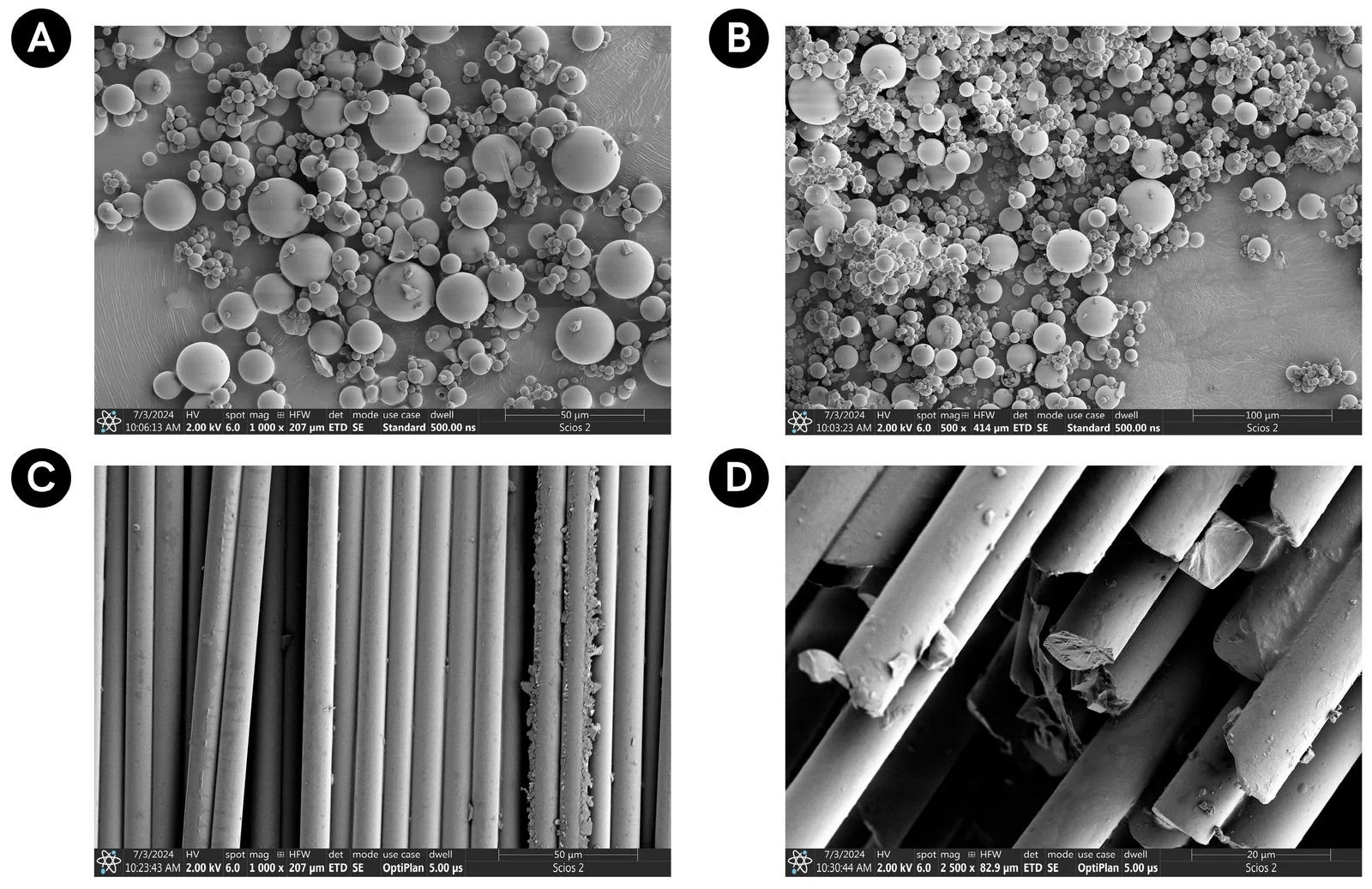
Representative SEM micrographs of the glass fillers used in this study: (**A**,**B**) hollow glass spheres and (**C**,**D**) chopped glass fibers.

Representative SEM micrographs of the as-received glass fillers are shown in Figure 1. The images illustrate the distinctly different reinforcement geometries investigated in this study, namely approximately spherical hollow glass particles and high-aspect-ratio chopped glass fibers. Quantitative particle-size statistics derived from the segmented SEM images of the composites are reported separately in Section 3.4.

### 2.2. Experimental Design and Preparation of Blends and Composites

The unfilled PHBV + PBAT + TPS blend was used as the reference matrix for all composite series. It was prepared using the same extrusion, pelletizing, drying, and injection-molding procedure as the glass-filled composites, but without the addition of glass fibers or glass spheres. Thus, the effect of the fillers was evaluated by comparing each composite with the identically processed ternary matrix rather than with the neat constituent polymers. This experimental design made it possible to distinguish the intrinsic phase behavior of the PHBV + PBAT + TPS matrix from the additional effects associated with filler geometry, filler content, and polymer–glass interfacial area.

The PHBV, PBAT, and TPS raw materials were processed without an additional pre-drying step immediately before melt compounding, and their moisture content was not independently measured before extrusion. Vacuum degassing was applied during twin-screw extrusion to assist in the removal of volatile components, including residual moisture.

The reference blend and the corresponding glass-filled composites were prepared by melt compounding using a ZSK026MC18 twin-screw extruder (Coperion, Stuttgart, Germany). For the unfilled matrix, PHBV, PBAT, and TPS granules were dosed simultaneously into the main feed zone and compounded under the temperature profile shown in Table 3. For the composite formulations, the polymer components were introduced through the main feed, whereas glass fibers or glass spheres were added using a side feeder in zone IV. During melt compounding, the screw speed was approximately 420–450 rpm, and the material throughput was approximately 30–37 kg × h^−1^. These parameters were adjusted within the stated ranges to maintain stable processing as filler type and content changed. The pressure of the molten material immediately upstream of the extrusion die/head was approximately 4.2 MPa. Extrusion residence time was not recorded during the original processing campaign and was therefore not estimated retrospectively. Each formulation (REF, GS-2, GS-4, GS-10, GS-15, GF-2, GF-4, GF-10, and GF-15) was prepared as a single compounding batch. Thus, the experimental campaign did not include independent processing-batch replication. All samples had the same polymer-matrix composition and were processed using the same processing sequence and temperature program. However, screw speed and material throughput varied within the ranges to maintain stable melt compounding as filler type and content changed.

The extruded strands were cooled using a water–air-cooling system and pelletized with a rotary knife. The obtained pellets were dried at 60 °C for 8 h before injection molding.

Test specimens were produced by injection molding using a Victory 200/50 injection-molding machine (ENGEL, Schwertberg, Austria). The cylinder/nozzle temperature profile was 155/155/160/160 °C. The injection pressure was 82–84 bar, holding pressure was 30 bar, plasticization/back pressure was 9 bar, screw speed was 160 rpm, and injection speed was 60 mm × s^−1^. Injection and holding times were 1.20–1.40 s and 25 s, respectively. The plasticization time was approximately 9 s. The cooling time was 25 s at a mold temperature of 40 °C, and a total injection-molding cycle was approximately 55 s. No deliberate control of filler orientation was applied during injection molding; therefore, any preferred fiber alignment observed in the final specimens was considered consistent with flow-induced orientation during processing.

The relative composition of the PHBV + PBAT + TPS matrix was kept constant in all formulations at 49.75 wt.% PHBV, 24.875 wt.% PBAT, 24.875 wt.% TPS, and 0.50 wt.% calcium stearate. The composites contained 2, 4, 10, or 15 wt.% of either glass spheres or glass fibers, where the filler mass fraction was defined relative to the total composite mass.

The nominal filler volume fractions were calculated from the corresponding mass fractions according to Equation (1):

(1) $$V_{f}\;=\;\frac{\frac{w_{f}}{\rho_{f}}}{\left[\frac{w_{f}}{\rho_{f}}\;+\;\frac{w_{m}}{\rho_{m}}\right]}$$

where *V_f_* is the filler volume fraction; *w_f_* and *w_m_* are the mass fractions of filler and matrix in the total composite formulation, respectively; and *ρ_f_* and *ρ_m_* are their corresponding densities.

Because the density of the unfilled matrix was not determined experimentally, its theoretical density was calculated from the nominal composition according to the reciprocal rule of mixtures, Equation (2):

(2) $$\rho_{m}\;=\;\frac{1}{\sum \frac{w_{i}}{\rho_{i}}}$$

Densities of 1.25, 1.21, 1.35, and 1.12 g × cm^−3^ were used for PHBV, PBAT, TPS, and calcium stearate, respectively. The densities of PHBV, PBAT, and TPS were obtained from the corresponding technical data sheets, whereas a literature value was used for calcium stearate [23]. The resulting theoretical density of the PHBV + PBAT + TPS + calcium-stearate matrix was 1.262 g × cm^−3^. Densities of 1.10 and 2.50 g × cm^−3^ were used for the hollow glass spheres and glass fibers, respectively. For the hollow glass spheres, the supplier-reported density of 1.10 g × cm^−3^ was treated as the effective density of the complete hollow particle. Consequently, the calculated filler volume fraction represents the nominal external geometric particle volume rather than the volume of solid glass forming the bead walls. The resulting compositions and corrected filler volume fractions are summarized in Table 4.

### 2.3. Microstructural Characterization and Deep-Learning Segmentation

The dispersion of glass fillers within the PHBV + PBAT + TPS matrix was examined using an ultra-high-resolution analytical dual-beam FIB-SEM instrument (Scios2 DualBeam, Thermo Fisher Scientific, Waltham, MA, USA) operated at an accelerating voltage of 5 kV. Observations were performed on cryogenic fracture surfaces at magnifications ranging from 250× to 5000×. For high-resolution imaging, the samples were coated with a conductive Au layer of approximately 5 nm using an EM ACE600 high-vacuum sputter coater (Leica, Wetzlar, Germany).

Automated identification of glass fibers and hollow glass spheres in SEM micrographs was performed using deep-learning instance-segmentation models from the Mask R-CNN family implemented in the Detectron2 framework (Meta AI Research, CA, USA) [17]. Separate detectors were trained for the two reinforcement types to account for their different morphologies. Separate training and validation datasets were prepared for each reinforcement class. For the glass fibers, 26 SEM images containing 90 manually annotated instances were used for training and eight independent images containing 34 instances were used for validation. For the hollow glass spheres, 15 SEM images containing 53 manually annotated instances were used for training, and eight independent images containing 37 instances were used for validation. The annotations were prepared in COCO format by manually delineating the contours of individual fibers or spheres. Images enriched in reinforcement instances were used only for model annotation and training and were not used as a selection criterion for the quantitative stereological dataset.

Both detectors used a ResNet-50 backbone with a Feature Pyramid Network (FPN), pre-trained on the COCO dataset. For the glass fibers, a Cascade Mask R-CNN architecture was used, whereas for the hollow glass spheres, a Mask R-CNN architecture was applied. Augmentation comprised resizing of the shortest image edge; horizontal flipping; and variation in brightness, contrast, and saturation. Rotational augmentation was not applied, because it could artificially alter the orientation characteristics of the anisotropic glass fibers. Training was performed for 5000 iterations using stochastic gradient descent with a base learning rate of 0.002, a batch size of two images with gradient accumulation over four steps, 200 warm-up iterations, and mixed-precision computation on a CUDA-enabled GPU using Detectron2 v0.6. Inference for the quantitative analysis was run with an explicit detection score threshold of 0.15, which overrides the default value of 0.20 stored in the saved configuration files; the COCO metrics reported in Table 5 were computed at the conventional evaluation threshold of 0.05.

Segmentation quality was assessed both visually, by inspecting prediction overlays, and quantitatively using COCO mask instance-segmentation metrics computed on the held-out validation sets at the conventional evaluation score threshold of 0.05. The quantitative validation results are summarized in Table 5. In addition, object-count validation was performed by comparing the number of automatically detected objects with the number of manually annotated objects on each validation image. The glass-fiber detector recovered 33 of 34 annotated objects (mean absolute error, 0.62 objects per image; count recall, 97.1%), whereas the hollow-glass-sphere detector recovered 26 of 37 annotated objects (mean absolute error, 1.62 objects per image; count recall, 70.3%). The implications of the lower detection recall of the hollow-glass-sphere detector for the segmentation-derived descriptors are addressed in Section 4.5.

In addition, automatically obtained size measurements were compared directly with manual annotations on the validation images. Detected objects were matched one-to-one with manually annotated objects at a mask IoU of at least 0.50, and the same size descriptors were calculated from the manual and automatic masks of each matched object. For 19 matched glass-fiber instances, the equivalent diameter differed from the manual value by −0.07 µm on average, with a mean absolute deviation of 0.41 µm (3.9%) and a Pearson correlation coefficient of r = 0.973. For 19 matched hollow-glass-sphere instances, the corresponding values were +0.22 µm, 0.71 µm (8.1%) and r = 0.950. The complete manual-versus-automatic comparison is provided in Supplementary Table S1.

To improve transparency and reproducibility, representative raw SEM images, manual annotations, and segmentation overlays are provided as Supplementary Materials (Figure S2), and the complete training and inference configuration of both detectors is provided in Supplementary Table S2. The trained weights of the two detectors (model_final_fibers.pth, Cascade Mask R-CNN; and model_final_spheres.pth, Mask R-CNN), together with their Detectron2 v0.6 configuration files and COCO-format manual annotations, are available from the corresponding author upon request; the weight files are not deposited with the article, because of their size (547 and 335 MB, respectively).

Separate sets of injection-molded specimens were produced for the individual test geometries. One specimen per formulation was selected for SEM examination. Quantitative SEM fields were acquired at randomly selected locations on the cryogenically fractured surface of the selected specimen. All quantitative stereological analyses were performed at a single, constant imaging scale. Each micrograph had a horizontal field width of 570 µm and a resolution of 1536 × 1094 pixels, corresponding to a calibration of 0.371 µm pixel^−1^ and an analyzed area of 2.314 × 10^5^ µm^2^ per field. The analysis included 4, 5, 5, and 5 SEM fields for GF-2, GF-4, GF-10, and GF-15, respectively, and 3, 5, 5, and 5 fields for GS-2, GS-4, GS-10, and GS-15, respectively. These fields contained 60, 92, 131, and 136 segmented fiber objects and 58, 44, 82, and 194 segmented hollow-glass-sphere objects, respectively. The quantitative fields therefore represent random spatial sampling within one molded specimen per formulation. Accordingly, the resulting stereological descriptors are interpreted as comparative two-dimensional measures of the analyzed fields rather than as independent specimen-level estimates of the complete three-dimensional filler distribution.

All segmentation and stereological measurements were performed on the original, uncompressed SEM image files. Before analysis, image calibration, scale verification, and visual quality control were carried out to ensure that filler–matrix boundaries were sufficiently resolved. Areas affected by charging, contamination, severe blur, poor focus, or insufficient contrast were excluded from quantitative analysis. The quality of the predicted segmentation masks was additionally verified by inspecting overlays of the masks on the original SEM images. The SEM figures included in the manuscript were prepared from the original high-resolution image files. It should also be noted that the segmentation workflow was used to quantify filler morphology and dispersion descriptors, such as projected size, center-to-center spacing, edge-to-edge distance, the nearest-neighbor index R, and specific polymer–glass interfacial area. It was not intended to quantify sub-resolution interfacial micropores or nanoscale defects.

For each segmented object, the centroid position and projected area were determined. For hollow glass spheres, the equivalent external diameter, D, was calculated from the projected area according to Equation (3):

(3) $$D=\;\sqrt{\frac{4A}{\pi }}$$

where *A* is the projected area of the segmented object. For glass fibers, the characteristic diameter was estimated as the minor axis of the best-fit ellipse fitted to the segmented object.

For each hollow-glass-sphere composition, the particle-size distribution was characterized using all segmented particles included in the quantitative analysis. The number of analyzed particles, mean ± standard deviation, median, minimum, maximum, and D10, D50, and D90 values are reported in Table 10. The characteristic diameter D used for the hollow glass spheres in Table 9 and in the subsequent stereological calculations corresponds to the composition-specific mean equivalent external diameter. For glass fibers, the characteristic diameter was estimated as the minor axis of the best-fit ellipse fitted to the segmented object.

The same best-fit ellipses were used to quantify the orientation of the glass fibers relative to the SEM section plane. Assuming cylindrical fiber geometry, a fiber intersected by the section plane at an angle θ to the section normal produces an elliptical profile for which the ratio of the minor to major axis corresponds to cosθ. The out-of-plane angle was therefore calculated as θ = arccos(minor/major) from the best-fit ellipse. Two limitations of this approach must be stated. First, the median θ obtained in this way is calculated directly from the observed sectional profiles and is not corrected for orientation-dependent sectioning bias, which over-samples fibers lying close to the section normal. Second, and more restrictive, the accessible range of θ is bounded by the largest section aspect ratio that can be resolved: the maximum measured major-to-minor axis ratio was 4.13, corresponding to θ ≤ 76°, so fibers lying close to the section plane are absent from the measured population. The orientation-tensor component a_zz_ = ⟨cos^2^θ⟩ was calculated using a 1/cosθ weighting to compensate for sectioning bias, but this weighting cannot compensate for the truncated angular range. Accordingly, a_zz_ was compared with the value expected for an isotropic three-dimensional population sampled over the same restricted range, a_zz_ = 0.43, rather than with the value of 1/3 that applies only when the full range from 0° to 90° is accessible.

The centroid coordinates obtained from the segmentation masks were used to determine the measured center-to-center distance, *C2C_measured_*, between neighboring reinforcement objects. Nearest-neighbor center-to-center and edge-to-edge distances were calculated separately within each SEM field. The values reported for each composition correspond to the mean ± standard deviation across the analyzed fields. Coordinates from different SEM fields were not pooled when determining nearest neighbors because objects located in separate fields do not belong to the same spatial neighborhood.

In addition, a theoretical center-to-center distance, *C2C_theoretical_*, was estimated for a random filler distribution at the same reinforcement volume fraction and characteristic filler size. This value was calculated using the stereological relation, Equation (4):

(4) $$L\approx D[\sqrt[{3}]{\frac{\pi }{6V_{V}}]}$$

where *D* is the characteristic filler diameter, and *V_V_* is the reinforcement volume fraction [19]. This theoretical value was used as a reference for assessing dispersion quality rather than as an exact description of the real microstructure. Because the hollow glass spheres exhibit a finite particle-size distribution, the theoretical three-dimensional *C2C* value represents an idealized compositional reference based on the composition-specific mean equivalent external diameter. It should not be interpreted as an exact representation of the complete polydisperse three-dimensional particle distribution.

To evaluate the spatial arrangement of the segmented filler objects using a dimensionally consistent two-dimensional descriptor, a nearest-neighbor index *R* was calculated separately for each SEM field as follows (5):

(5) $$R=\frac{{\bar{C2C}}_{measured}}{0.5/\sqrt{N_{A}}}$$

where ${\bar{C2C}}_{measured}$ is the mean measured nearest-neighbour center-to-center distance within a given SEM field, and *NA* is the areal number density of segmented objects in that field. The denominator $0.5/\sqrt{N_{A}}$ represents the expected mean nearest-neighbor distance for a random planar arrangement at the same areal number density. Values of *R* close to unity indicate a random-like planar arrangement, values below unity indicate clustering, and values above unity indicate mutual exclusion or a more regular arrangement. The index is reported as mean ± standard deviation across the analyzed fields of each composition. No edge correction was applied; consequently, *R* may be slightly biased upward.

The theoretical three-dimensional center-to-center distance calculated from Equation (4) was retained only as an idealized compositional reference and was not used to calculate *R*, because the measured SEM distances and the theoretical relation represent two-dimensional and three-dimensional quantities, respectively.

Values close to unity indicate a random-like distribution, whereas markedly lower values may indicate local clustering or reduced inter-object spacing. In the case of fibers, deviations from unity may also result from elongated filler geometry and flow-induced orientation during injection molding. Edge-to-edge distances, E2E, were also determined to assess the degree of close contact between neighboring objects and the possible occurrence of local agglomeration.

The specific external polymer–glass interfacial area per unit composite volume, (*S_V_*), was calculated to quantify the geometrically available reinforcement–matrix contact area. For the hollow glass spheres, $S_{V}=\frac{6V_{V}}{D}$ was used, whereas for long cylindrical fibers, $S_{V}=\frac{4V_{V}}{D}$ was applied, neglecting the comparatively small contribution of the fiber end surfaces. For the hollow glass spheres, *V_V_* represents the nominal external geometric particle volume fraction calculated using the supplier-reported effective density of the complete hollow particle, while *D* represents the composition-specific mean equivalent external diameter determined from the segmented SEM images. Consequently, the calculated *S_V_* for the hollow glass spheres describes only the external bead surface geometrically available for contact with the polymer matrix and does not include the internal surface of the hollow particles. For the glass fibers, *D* denotes the composition-specific characteristic fiber diameter determined from the segmented SEM images. All calculations were performed using the corrected filler volume fractions and the characteristic diameters obtained from the same regenerated analysis dataset.

### 2.4. Density Measurements

Skeletal density was determined by helium pycnometry using an AccuPyc II 1340 (Micromeritics, Norcross, GA, USA). The sample volume is measured using helium as a gas permeating through the sample pores. The test was performed in accordance with the ASTM D6226 [24]/ISO 1183 standard [25]. The test was performed using a standard measuring cell with dimensions φ =1.82 cm; h = 3.93 cm; and measuring chamber filling pressure—1.34 bar.

### 2.5. Differential Scanning Calorimetry (DSC) Analysis

Differential scanning calorimetry (DSC) was performed according to ISO 11357 [26,27] using a DSC 213 Polyma calorimeter (Netzsch, Selb, Germany). The measurements were conducted under a nitrogen atmosphere at a gas flow rate of 50 mL × min^−1^. Heating and cooling scans were performed at a constant rate of 10 °C × min^−1^ over the temperature ranges of 20–300 °C and 300–20 °C, respectively. An empty aluminum crucible was used as the reference.

The recorded thermograms were analyzed to determine the glass-transition temperature (T_g_), melting temperature (T_m_), crystallization temperature (T_c_), melting enthalpy (ΔH_m_), and crystallization enthalpy (ΔH_c_). The melting and crystallization enthalpies were obtained by integrating the corresponding endothermic and exothermic peaks. The degree of crystallinity was estimated from the measured melting enthalpy using the theoretical melting enthalpy of 100% crystalline PHBV. Because the investigated matrix contained several polymer components with different thermal responses, the calculated value is referred to as the DSC-based apparent crystallinity.

DSC was used to compare the thermal behavior of PHBV, PBAT, TPS, the unfilled PHBV + PBAT + TPS blend, and the corresponding glass-filled composites, and to assess the influence of glass-filler geometry and content on phase transitions and crystalline organization of the polymer matrix.

### 2.6. Melt Flow Rate Measurements

The melt mass-flow rate (MFR) was determined using a ZwickRoell Mflow extrusion plastometer (ZwickRoell, Ulm, Germany) in accordance with ISO 1133 [28] (procedure technically equivalent to ASTM D1238 [29] under the selected conditions). Measurements were performed at 190 °C under a nominal load of 2.16 kg. The molten material was extruded through a standard die under controlled temperature and load, and the mass of extrudate collected over a defined time interval was converted into the MFR, expressed in g × 10 min^−1^. The MFR values were used as comparative indicators of the apparent melt flowability of the unfilled PHBV + PBAT + TPS blend and the corresponding glass-filled composites under identical test conditions.

### 2.7. X-Ray Diffraction (XRD) Analysis

XRD data were collected using a D8 Advance Eco diffractometer (Bruker, Karlsruhe, Germany) in Bragg–Brentano geometry with Ni-filtered Cu Kα radiation (λ = 1.5406 Å), operated at 40 kV and 25 mA. Diffraction patterns were recorded at room temperature over the 2θ range of 5–50°, with a step size of 0.01° and a counting time of 1.5 s per step, corresponding to a scanning speed of 0.4° × min^−1^. XRD data were collected for one representative injection-molded specimen per formulation (*n* = 1). The fitting uncertainty of the deconvolution procedure was quantified using Gaussian error propagation from the parameter covariance matrix (Supplementary Table S3).

**Figure 2:**
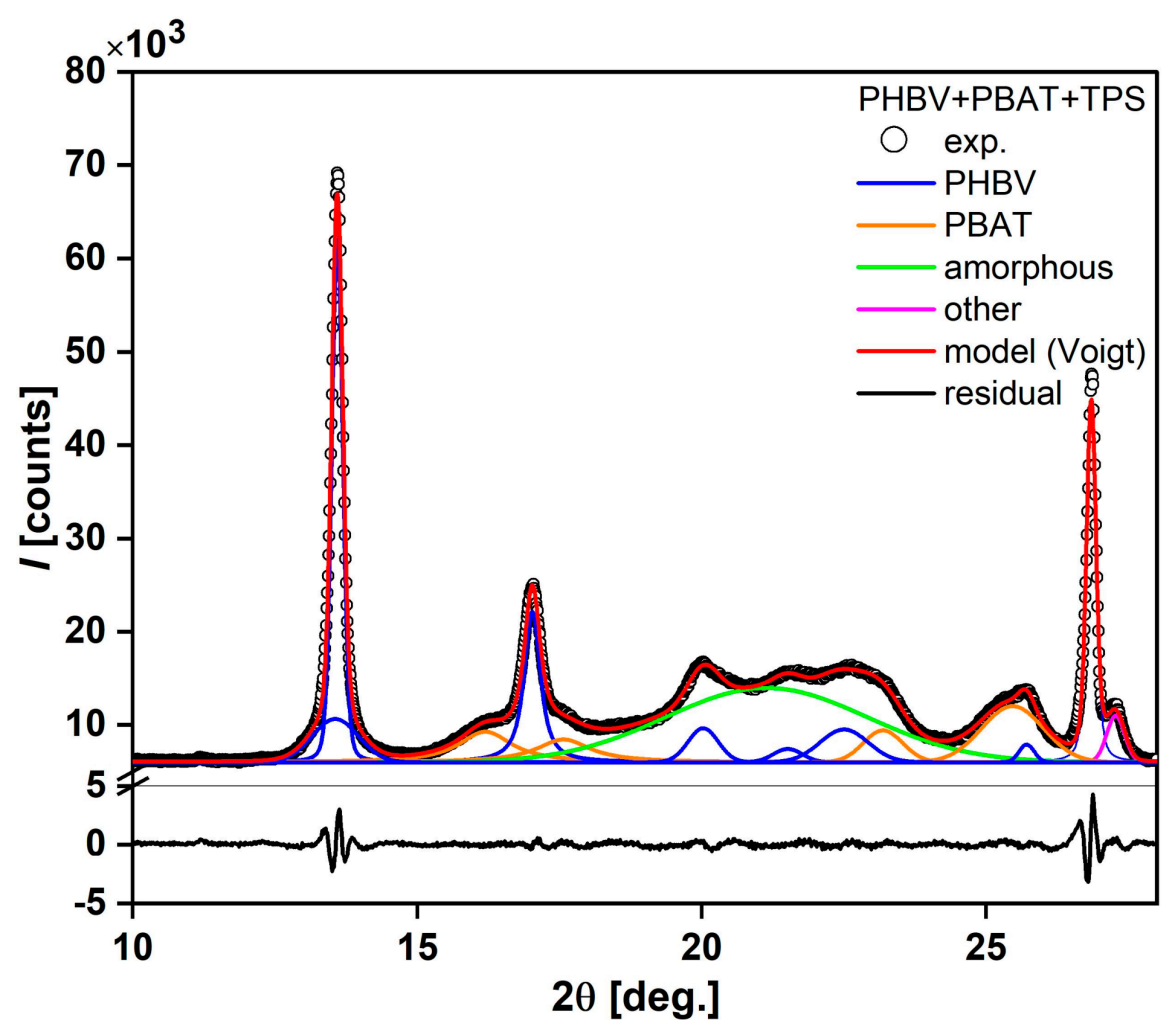
Representative XRD profile deconvolution for the base PHBV + PBAT + TPS blend demonstrating the curve-fitting method used for crystallinity determination. The experimental data (*open circles*) were fitted with a cumulative model (*red line*) comprising discrete crystalline reflections for PHBV (*blue line*) and PBAT (*orange line*), along with a broad amorphous halo (*green line*). The peak at 2θ ≈ 27.3° corresponds to the unidentified reflection of trace impurities from inorganic additives (*magenta line*). The residual curve (*bottom black line*) indicates the goodness of fit.

The diffraction profiles were examined for crystal structure identification and assessment of the degree of crystallinity. The results were pre-processed using DIFFRAC.EVA 4.1.1 software to subtract the instrumental background (linear baseline correction). Subsequently, the degree of crystallinity was determined using the curve-fitting method, following the procedure established by Hindeleh and Johnson [30]. Diffractograms were decomposed into separate crystalline reflections and amorphous halos in Fityk 1.3.1 software using Voigt profile functions (Figure 2). The average crystallite size was calculated using the Scherrer equation, assuming a shape factor of K = 0.9 [31], after correcting for instrumental line broadening determined using the NIST SRM 1976b standard [32]. The crystallite size, *D*, was calculated specifically for the PHBV phase using the (110) reflection. This reflection was selected because it is the most intense peak that remains well-resolved from the amorphous halo and overlapping reflections of the secondary phases (PBAT/TPS) and is less susceptible to the preferred orientation effects observed for the (0k0) basal reflections, thus providing a more representative estimation of the crystal dimension.

The crystallinity determined by XRD is referred to as an XRD-based crystallinity index because it is obtained from the ratio of crystalline diffraction peaks to the total scattered intensity and may be affected by peak overlap, background subtraction, and preferred orientation. For the glass-filled materials, this quantity is referred to as the composite-level XRD-based crystallinity index because it represents the relative crystalline scattering contribution of the complete composite rather than the intrinsic crystallinity of an individual polymer phase.

Crystalline-peak assignment in the PHBV + PBAT + TPS blend and the corresponding composites was based on comparison with the diffraction patterns of the neat PHBV, PBAT, and TPS components recorded under the same experimental conditions. Reflections occurring at positions characteristic of neat PHBV and PBAT were assigned to the respective crystalline phases and fitted separately, whereas the broad non-crystalline contribution was represented by the amorphous halo. Although weak residual crystalline reflections were detected for neat TPS, no distinct TPS-related reflections could be resolved in the ternary blend; consequently, a separate TPS crystalline contribution was not introduced into the peak-area analysis of the blend and composites.

Three distinct structural descriptors were considered in the XRD analysis. The composite-level XRD-based crystallinity index represents the relative contribution of fitted crystalline scattering to the total diffraction profile. The Scherrer-derived value obtained from the PHBV (110) reflection represents an apparent coherent-domain dimension normal to the corresponding lattice planes and should not be interpreted as the total crystalline fraction. Preferred orientation was assessed independently using the texture index derived from the area ratio of the PHBV (020) and (110) reflections. These quantities therefore describe different aspects of crystalline structure and were interpreted separately.

The XRD–calculated crystallinity index ${(X}_{c}^{XRD})$ was determined as the ratio of the total area of crystalline peaks to the total scattering area [33], according to Formula (6):

(6) $$X_{C}^{XRD}=\frac{\sum A_{c}}{\sum \left(A_{c}+A_{a}\right)}\cdot 100\%$$

where *A_c_* is the integrated area of the crystalline peaks, and *A_a_* is the total integrated area of the amorphous halo across the studied patterns. Due to the strong preferred orientation of PHBV crystals in the studied samples, manifested by the anomalously high intensity of the (0k0) reflections (induced by shear flow during injection molding), the calculations required intensity correction. The (110) reflection was selected as an internal standard due to its lower sensitivity to planar orientation. The theoretical intensities of the textured (020) and (040) peaks were recalculated assuming an isotropic crystal distribution, using relative intensity ratios from the ICDD card 00-035-1340 ($I_{\left(020\right)}^{PHBV}/I_{\left(110\right)}^{PHBV}=1.30$, $I_{\left(040\right)}^{PHBV}/I_{\left(110\right)}^{PHBV}=0.21$). The corrected crystalline area was calculated according to Equation (7):

(7) $$A_{c}^{corr}=A_{c}^{raw}-\sum_{hkl\in \{(020),(040)\}}\left(A_{hkl}^{meas}-r_{hkl}\cdot A_{\left(110\right)}^{meas}\right)$$

where $r_{hkl}=\frac{I_{\left(hkl\right)}^{ICDD}}{I_{\left(110\right)}^{ICDD}}$, with $r_{020}=1.30$ and $r_{040}=0.21$. The corrected XRD-based crystallinity index was subsequently calculated as $X_{c}^{XRD,corr}=\frac{A_{c}^{corr}}{A_{c}^{corr}+A_{a}}\cdot 100\%$.

The deconvolution fits showed high coefficients of determination (R^2^ = 0.9916–0.9983). Propagation of the fitting errors from the parameter covariance matrix yielded an uncertainty of 0.8–1.2 percentage points in the XRD-based crystallinity index. A detailed sensitivity comparison between uncorrected and corrected crystallinity values, together with individual profile-fitting statistics for all analyzed patterns, is provided in Supplementary Table S3.

### 2.8. Mechanical Testing

#### 2.8.1. Static Tensile Test

The static tensile test was performed using an MTS 858 Table Top System (MTS Systems Corporation, Eden Prairie, Minnesota) servo-hydraulic testing machine (measuring range up to 25 kN) equipped with an ARAMIS 3D 4M vision system for non-contact strain measurement. The test was conducted in accordance with EN ISO 527-1 [34] on type 1A specimens (dimensions as per EN ISO 527-2 [35]). To enable data acquisition with the vision system, a black-and-white stochastic pattern was applied to the surface of the specimens to capture geometric changes.

The experiments were conducted with samples of 75 mm gauge at a strain rate of 2.2 × 10^−4^ × s^−1^, whereas the test was subsequently continued at 1.25 × 10^−2^ × s^−1^ to determine the maximum tensile stress and strain at break. Each composition was tested on five specimens. The resulting strength parameters were averaged, and the standard deviations were calculated.

#### 2.8.2. Charpy Impact Test

The impact test was carried out using the Charpy method in accordance with ISO 179-1:2010 standard [36]. The pendulum hammer HIT5 (Zwick Roell, Ulm, Germany) was used. Dimensions of rectangular beam specimen were 80 mm × 10 mm × 4 mm (*l*, *b*, and *h*) in accordance with EN ISO 179-1:2010 standard [36]. Experiments were conducted on samples with and without notch, supported at both ends. The measurement was carried out by edge striking the center of the beam between the supports. The tests were performed using both notched and unnotched specimens, with *n* = 4 for each specimen configuration and formulation. The results are reported as mean ± standard deviation.

### 2.9. Replication and Data Treatment

The level of replication differed among the experimental methods. Each formulation was prepared as a single compounding batch; consequently, independent processing-batch replication was not available.

Tensile testing was performed using five independently tested molded specimens per formulation (*n* = 5), whereas Charpy testing included four unnotched and four notched specimens per formulation (*n* = 4 for each configuration). These mechanical results are reported as mean ± standard deviation and characterize within-batch specimen variability.

For MFR, three repeat determinations were performed using material from one sample per formulation. Density was determined for one specimen per formulation using ten repeated pycnometer measurement cycles. These measurements were treated as technical repeats rather than independent material replicates.

One DSC thermogram and one XRD pattern were acquired per formulation (*n* = 1). The corresponding results were therefore interpreted descriptively. The uncertainty associated with XRD profile deconvolution was evaluated separately by Gaussian error propagation from the fitting covariance matrix.

For SEM analysis, one molded specimen per formulation was examined, and 3–5 randomly selected fields were analyzed. The fields represent spatial subsampling within the selected specimen rather than independent specimen-level replication.

Because independent compounding batches were not produced for each formulation, no inferential statistical comparisons among formulations were performed. Differences among formulations are therefore described as comparative trends within the investigated processing campaign, and no claims of statistical significance are made.

## 3. Results

### 3.1. Thermal and Crystalline Characteristics of the Neat Components and Reference Matrix

**Figure 3:**
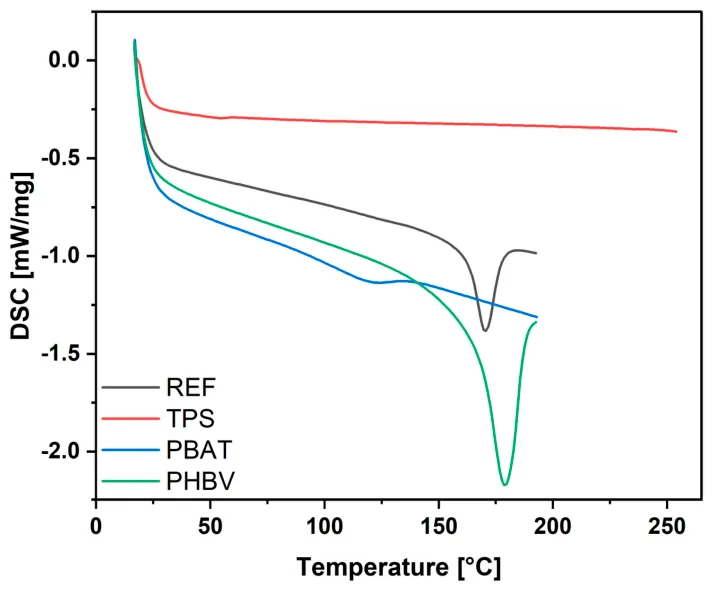
DSC thermograms of neat PHBV, PBAT, and TPS and the unfilled PHBV + PBAT + TPS reference blend.

The DSC thermograms of neat PHBV, PBAT, and TPS, together with that of the unfilled PHBV + PBAT + TPS blend, are presented in Figure 3. The corresponding melting enthalpies and DSC-based apparent crystallinity values are summarized in Table 6.

Neat PHBV exhibited a well-defined melting transition extending from approximately 168 to 187.5 °C, with a peak maximum at approximately 179 °C. Its measured melting enthalpy was 98.2 J × g^−1^, corresponding to a DSC-based crystallinity of 66.8%.

PBAT exhibited a lower-temperature melting transition in the range of approximately 94–133 °C, with a peak maximum at approximately 129 °C. The measured melting enthalpy was 9.2 J × g^−1^, corresponding to a DSC-based crystallinity of 8.1%. TPS did not exhibit a distinct melting peak under the applied measurement conditions and was therefore treated as an amorphous component in the DSC-based calculations.

The DSC thermogram of the unfilled PHBV + PBAT + TPS blend was dominated by the PHBV melting transition. The measured melting enthalpy of the blend was 33.4 J × g^−1^, corresponding to a DSC-based apparent crystallinity of 32.8%. A lower-temperature thermal contribution associated with PBAT was also observed, whereas no distinct TPS melting transition was detected.

**Figure 4:**
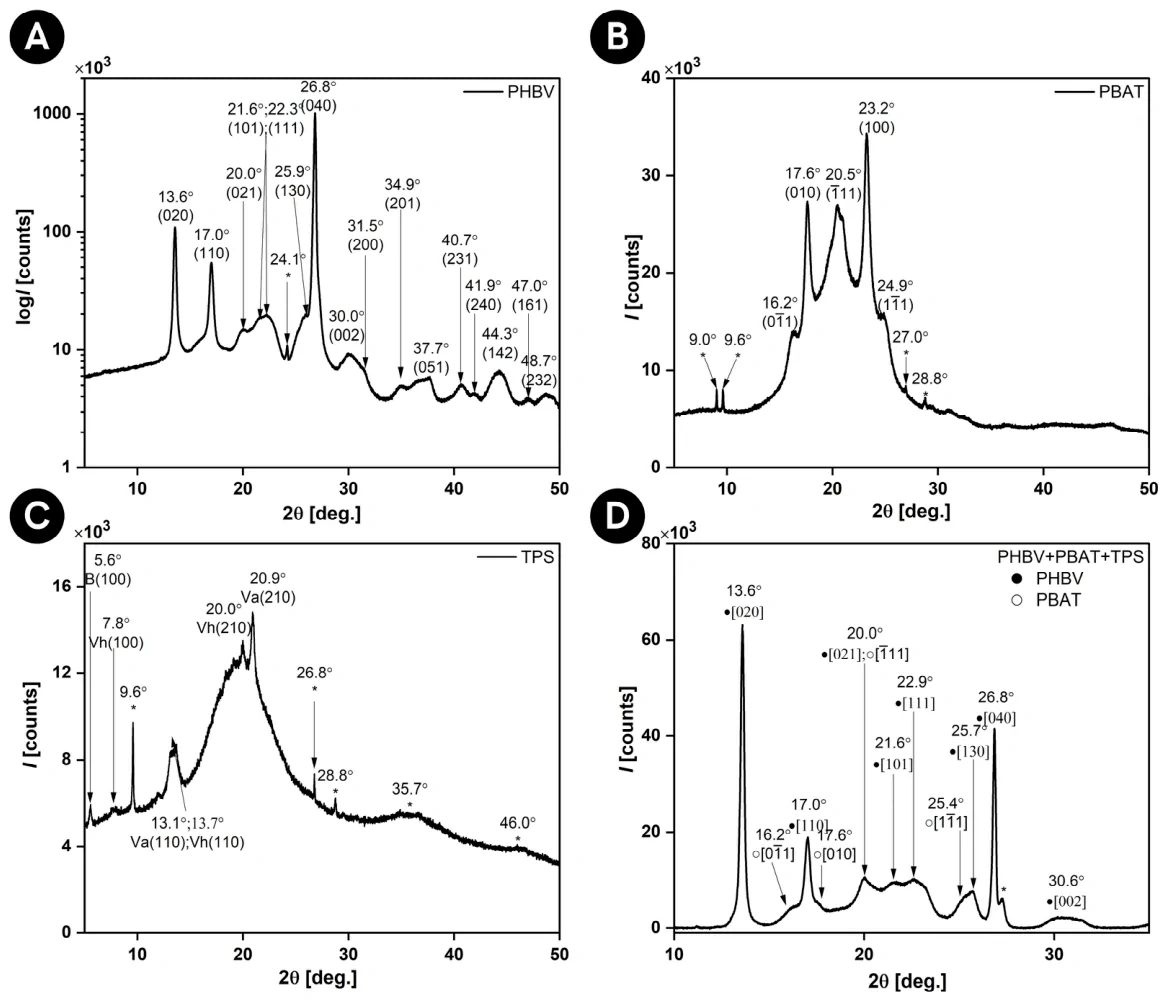
XRD patterns of neat (**A**) PHBV, (**B**) PBAT, (**C**) TPS, and (**D**) the unfilled PHBV + PBAT + TPS blend, with the identified crystalline reflections. Unidentified peaks and reflections associated with trace impurities are marked with an asterisk (*).

The XRD patterns of the neat components and the unfilled PHBV + PBAT + TPS blend are presented in Figure 4. The identified crystalline phases, calculated lattice parameters, and XRD-based crystallinity indices are summarized in Table 7.

Neat PHBV exhibited diffraction reflections characteristic of the orthorhombic α-PHB crystalline structure. Its corrected XRD-based crystallinity index was 77.4%. Differences in the relative intensities of selected PHBV reflections were also observed.

The PBAT diffraction pattern consisted of a broad amorphous contribution and crystalline reflections assigned to the butylene terephthalate-rich segments. The resulting XRD-based crystallinity index was 37.5%.

The TPS pattern was dominated by a broad amorphous halo. Weak diffraction reflections assigned to residual B-type starch structures, and hydrated and anhydrous V-type complexes were also detected. The XRD-based crystallinity index of TPS was 11.3%.

The diffraction pattern of the unfilled PHBV + PBAT + TPS blend was dominated by PHBV-related reflections, accompanied by weaker PBAT-related reflections and a broad amorphous contribution. No distinct crystalline reflections attributable to TPS were identified in the blend. No additional diffraction reflections indicating the formation of new crystalline phases were detected. The XRD-based crystallinity index of the unfilled PHBV + PBAT + TPS blend was 59.1%.

### 3.2. Filler-Induced Changes in Thermal Behavior and Crystalline Structure

**Figure 5:**
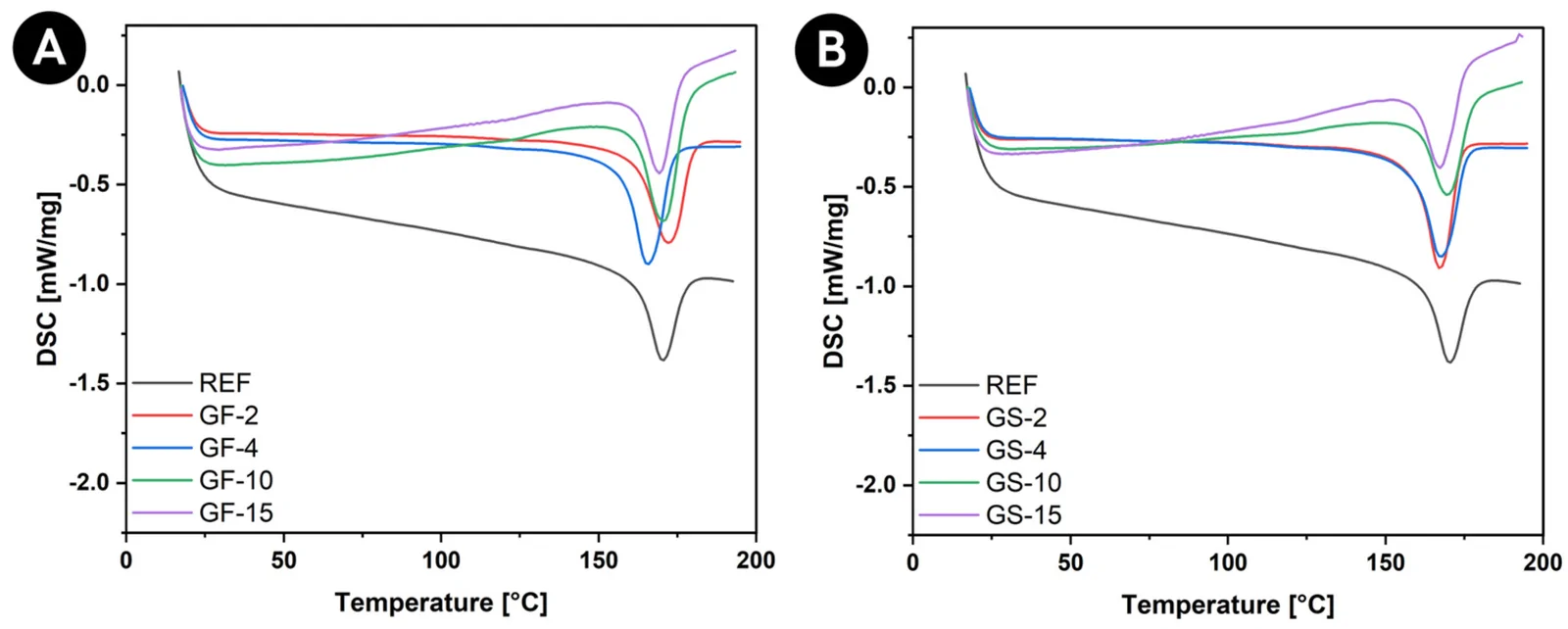
DSC thermograms of PHBV + PBAT + TPS composites containing different amounts of (**A**) glass fibers and (**B**) glass spheres.

The DSC thermograms of the PHBV + PBAT + TPS composites containing glass fibers and glass spheres are presented in Figure 5. In both composite series, the thermal response was dominated by the PHBV melting transition. The position and general shape of the main PHBV-related melting region remained similar across the investigated compositions. At higher filler contents, weak endothermic effects were also observed in the lower-temperature range corresponding to the PBAT melting transition identified for the neat polymer.

**Figure 6:**
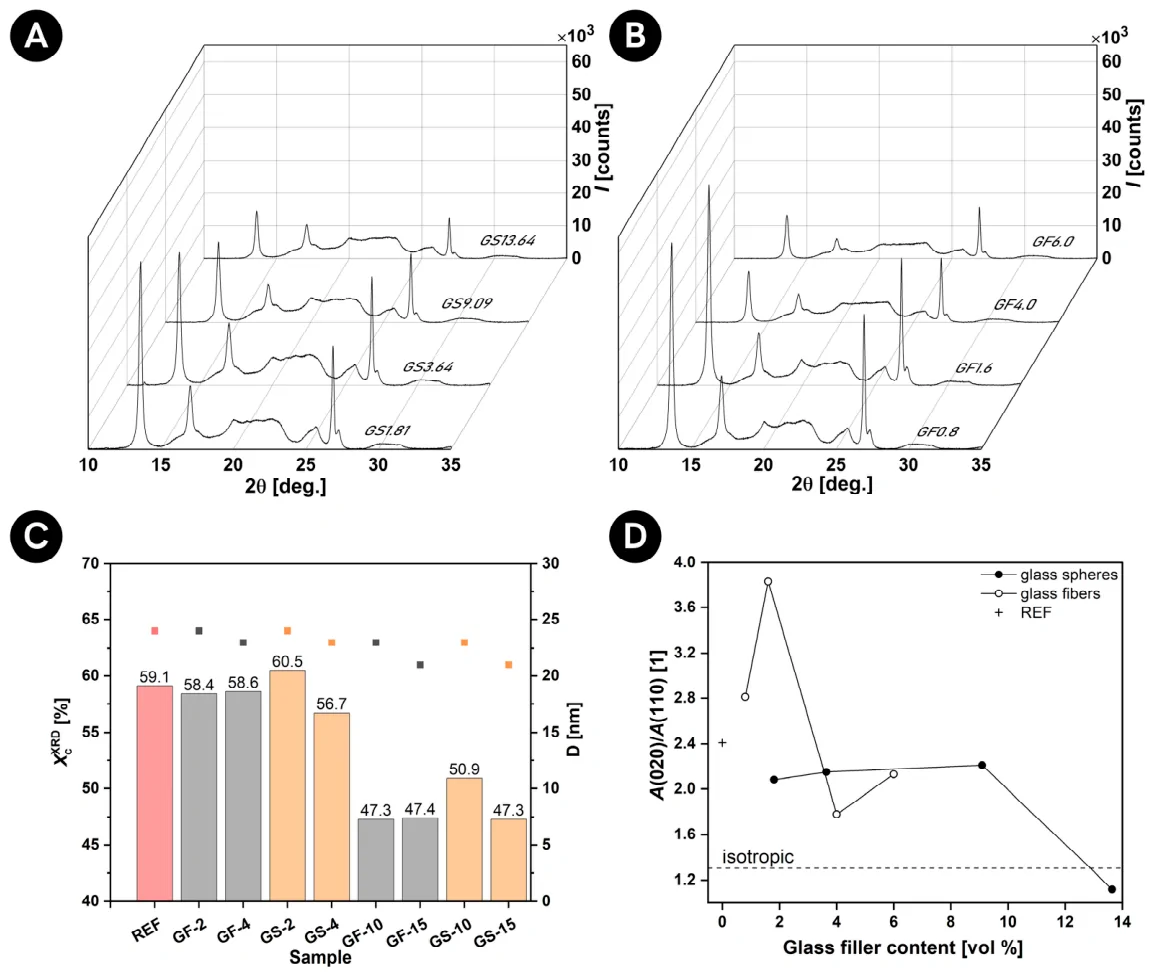
XRD analysis of PHBV + PBAT + TPS composites: Stacked diffraction patterns of samples containing (**A**) hollow glass spheres and (**B**) glass fibers; (**C**) composite-level XRD-based crystallinity index and Scherrer-derived apparent PHBV (110) coherent-domain size; and (**D**) texture index as a function of glass-filler content.

The XRD patterns of the composites containing glass spheres and glass fibers are shown in Figure 6A and Figure 6B, respectively. The characteristic diffraction reflections of the PHBV + PBAT + TPS matrix were retained in all investigated compositions. No additional crystalline reflections were detected after the incorporation of either glass fibers or glass spheres. Increasing filler content was accompanied by changes in the relative intensities of the polymer-related reflections and in the contribution of the broad non-crystalline background.

The composite-level XRD-based crystallinity indices and the apparent PHBV coherent-domain sizes estimated from the (110) reflection using the Scherrer equation are presented in Figure 6C. The unfilled PHBV + PBAT + TPS matrix exhibited an XRD-based crystallinity index of 59.1%. At the lowest hollow-glass-sphere content of 2.29%, the composite-level XRD-based crystallinity index increased slightly to 60.5%. At low glass-fiber contents, the values remained close to that of the unfilled reference matrix.

With increasing filler content, the composite-level XRD-based crystallinity index decreased in both composite series, reaching approximately 47% at the highest investigated filler contents. The apparent PHBV (110) coherent-domain size remained within approximately 21–24 nm across the investigated compositions.

The texture index, calculated from the area ratio of the PHBV (020) and (110) reflections, is presented in Figure 6D. The unfilled PHBV + PBAT + TPS matrix exhibited a texture index of approximately 2.4. In the glass-sphere-filled composites, the texture index remained relatively stable at low and intermediate filler contents and decreased at the highest sphere content. Greater variation in the texture index was observed in the glass-fiber-filled series.

### 3.3. Melt Flow Rate

The melt mass-flow rate values of the unfilled PHBV + PBAT + TPS matrix and the corresponding glass-filled composites are summarized in Table 8.

The unfilled PHBV + PBAT + TPS reference matrix exhibited an MFR of 13.6 g × 10 min^−1^. The incorporation of glass spheres produced a non-monotonic concentration-dependent response. At glass-sphere contents of 2.29 and 4.56 vol.%, the MFR increased to 18.8 and 26.8 g × 10 min^−1^, respectively. The highest value was therefore obtained for the composite containing 4.56 vol.% glass spheres. At higher sphere contents, the MFR decreased to 8.5 g × 10 min^−1^ at 11.31 vol.% and 7.6 g × 10 min^−1^ at 16.84 vol.%, with both values falling below that of the unfilled reference matrix.

A different trend was observed for the glass-fiber-filled composites. The MFR decreased progressively from 12.6 g × 10 min^−1^ at 1.02 vol.% glass fibers to 11.7 g × 10 min^−1^ at 2.06 vol.%, 6.5 g × 10 min^−1^ at 5.31 vol.% and 5.9 g × 10 min^−1^ at 8.18 vol.%. Thus, the lowest MFR among all investigated materials was recorded for the composite containing 8.18 vol.% glass fibers.

Overall, the glass-sphere- and glass-fiber-filled composites exhibited distinctly different MFR trends. The sphere-filled series showed an initial increase followed by a decrease at higher filler contents, whereas the fiber-filled series showed a progressive reduction in MFR with increasing reinforcement content.

### 3.4. Filler Distribution, Interfacial Area, and Composite Density

**Figure 7:**
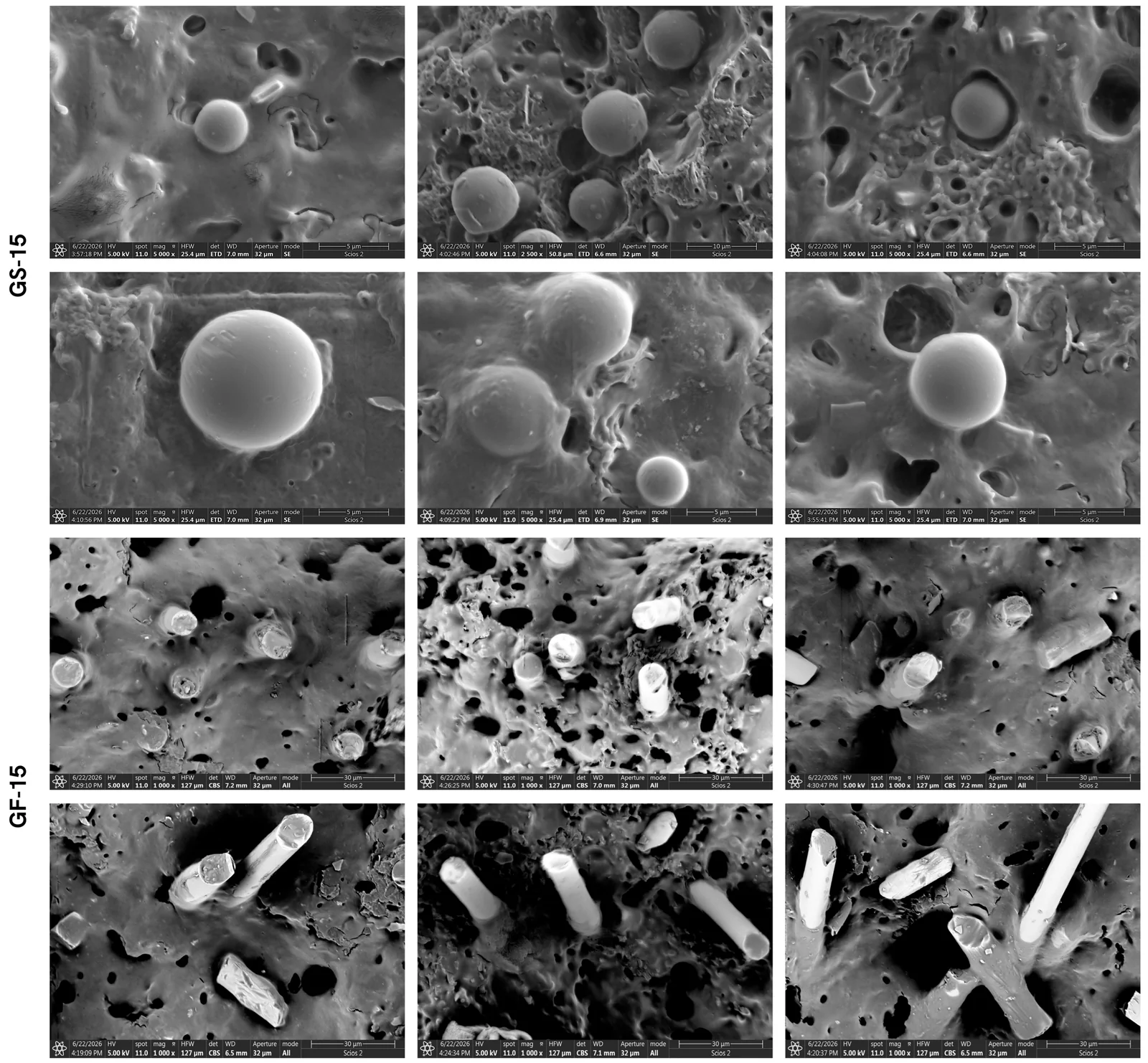
Representative SEM micrographs of cryogenically fractured PHBV + PBAT + TPS composites containing glass spheres and chopped glass fibers. The images show the distribution of the fillers and the matrix–filler morphology.

Representative SEM micrographs of the cryogenic fracture surfaces of the glass-sphere- and glass-fiber-filled composites are presented in Figure 7. Cryogenic fracture was used to examine the distribution of the fillers and the matrix–filler morphology while limiting deformation associated with mechanical loading.

Both reinforcement types were distributed throughout the PHBV + PBAT + TPS matrix. No pronounced filler-rich regions were observed in the analyzed fields of view. Glass spheres occurred predominantly as isolated particles or as locally neighboring objects surrounded by polymer-rich regions. Glass fibers were observed as elongated inclusions intersecting the fracture surface at different positions and orientations. Local variations in the distances between neighboring filler objects were visible in both composite series.

**Figure 8:**
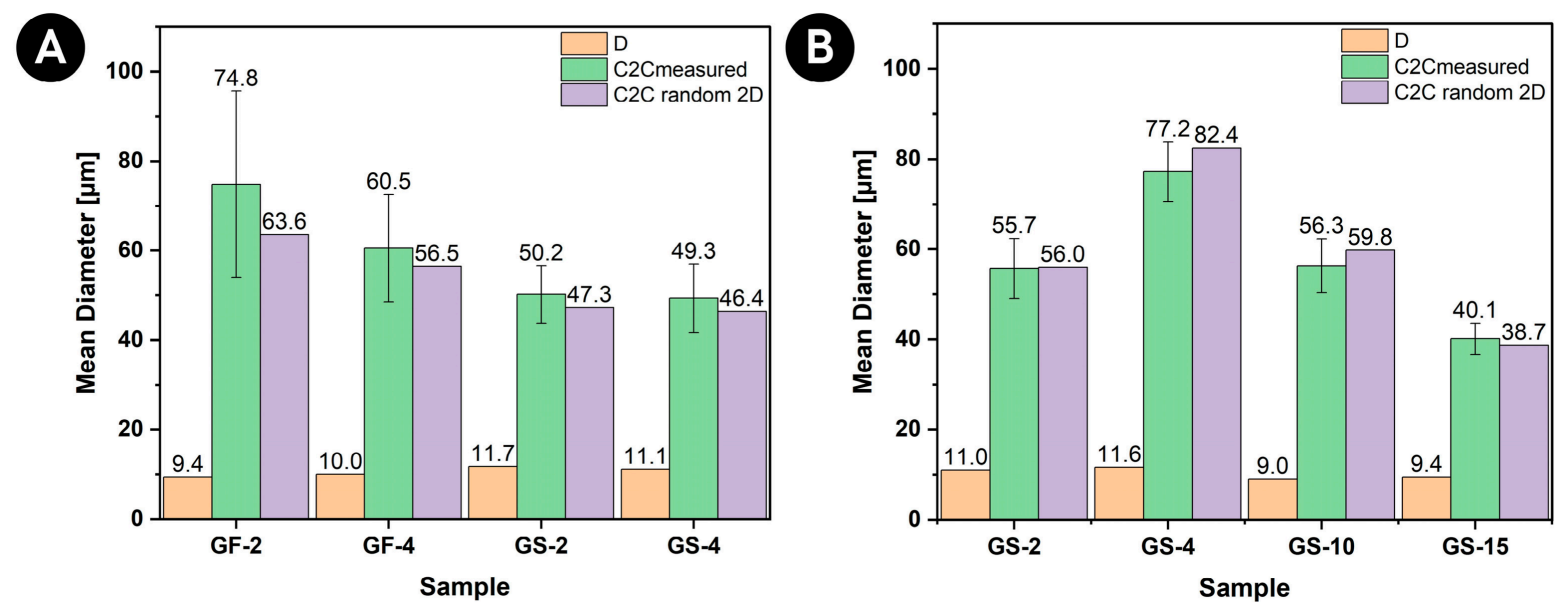
Segmentation-derived stereological parameters of PHBV + PBAT + TPS composites containing (**A**) glass fibers and (**B**) hollow glass spheres: Characteristic filler diameter, measured nearest-neighbor center-to-center distance (mean ± SD across SEM fields), and the corresponding random 2D center-to-center reference.

**Figure 9:**
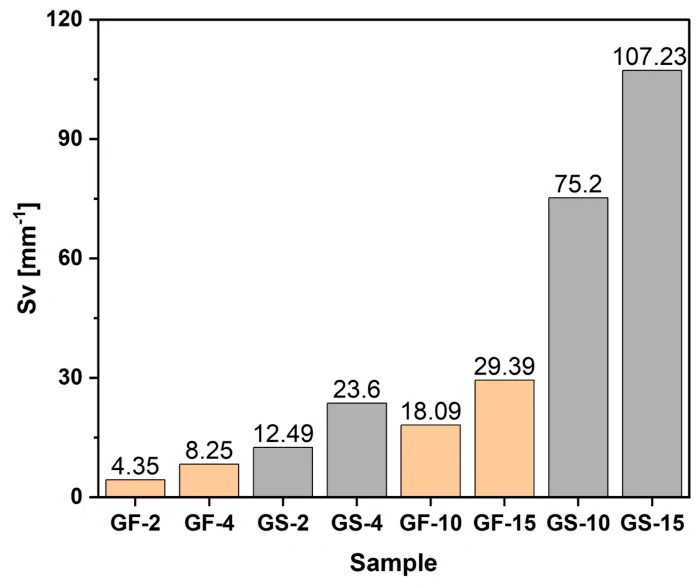
Specific external polymer–glass interfacial area as a function of glass-filler type and volume fraction.

The qualitative SEM observations were complemented by deep-learning-based segmentation. Representative raw SEM images, manual annotations, and segmentation overlays are provided in the Supplementary Materials, Figure S2. The segmentation-derived stereological descriptors are summarized in Table 9. The quantitative segmentation analysis included 58, 44, 82, and 194 hollow glass spheres for GS-2, GS-4, GS-10, and GS-15, respectively. The corresponding mean equivalent external diameters were 10.99 ± 3.61, 11.60 ± 4.89, 9.02 ± 3.20, and 9.42 ± 2.32 µm. Individual detected sphere diameters ranged from 5.23 to 28.61 µm across the analyzed compositions. Complete composition-specific particle-size distribution statistics are summarized in Table 10. The characteristic filler diameters, measured nearest-neighbor center-to-center distances, and corresponding random 2D center-to-center references are presented in Figure 8, whereas the specific external polymer–glass interfacial area is shown in Figure 9.

For the hollow-glass-sphere-filled composites, the measured nearest-neighbor center-to-center distance changed from 55.7 ± 6.7 µm at 2.29 vol.% to 40.1 ± 3.5 µm at 16.84 vol.%, while the corresponding edge-to-edge distance changed from 44.7 ± 7.0 to 30.7 ± 3.5 µm (mean ± standard deviation across SEM fields). The two-dimensional nearest-neighbor index R ranged from 0.94 to 1.04, remaining within approximately 6% of unity. Within the analyzed SEM fields, these values indicate no pronounced particle-rich clustering.

For the glass-fiber-filled composites, the measured nearest-neighbor center-to-center distance changed from 74.8 ± 20.8 µm at 1.02 vol.% to 49.3 ± 7.7 µm at 8.18 vol.%, and the corresponding edge-to-edge distance from 65.5 ± 20.8 to 38.1 ± 7.4 µm. The nearest-neighbor index R ranged from 1.06 to 1.16. The slightly supra-unity values are consistent with mutual exclusion of elongated sectioned objects rather than pronounced clustering. The field-to-field variability was greatest at the lowest fiber content.

The segmentation masks were additionally used to quantify the orientation of the glass fibers relative to the section plane. Across 419 segmented fibers, the uncorrected, section-derived median out-of-plane angle was 47.1°, against 43.3° expected for an isotropic three-dimensional population sampled over the same restricted angular range. After applying the 1/cosθ sectioning-bias correction, the orientation-tensor component, a_zz_, ranged from 0.38 to 0.44 across the four fiber-filled compositions, with a pooled value of 0.41, against 0.43 for an isotropic population over the same range. Neither descriptor departs from its isotropic reference in a direction or by a margin that would indicate preferred orientation, and both are essentially independent of fiber content.

The analysis therefore does not resolve a preferred fiber orientation in the examined sections. This is attributed to the restricted range of section geometries accessible in these micrographs rather than to a demonstrated absence of orientation in the molded material: highly oblique sections, which carry most of the information about fibers lying close to the section plane, are not captured, and the small projected size of the objects limits the precision with which the axis ratio can be determined.

The specific external polymer–glass interfacial area increased with filler content in both composite series. For the glass-fiber-filled composites, it increased from approximately 4.35 mm^−1^ at 1.02 vol.% fibers to 29.39 mm^−1^ at 8.18 vol.%. For the hollow-glass-sphere-filled composites, the corresponding value increased from approximately 12.49 mm^−1^ at 2.29 vol.% spheres to 107.23 mm^−1^ at 16.84 vol.%. Within the investigated composition ranges, the hollow-glass-sphere-filled composites therefore exhibited a substantially larger specific external polymer–glass interfacial area than the glass-fiber-filled composites.

**Figure 10:**
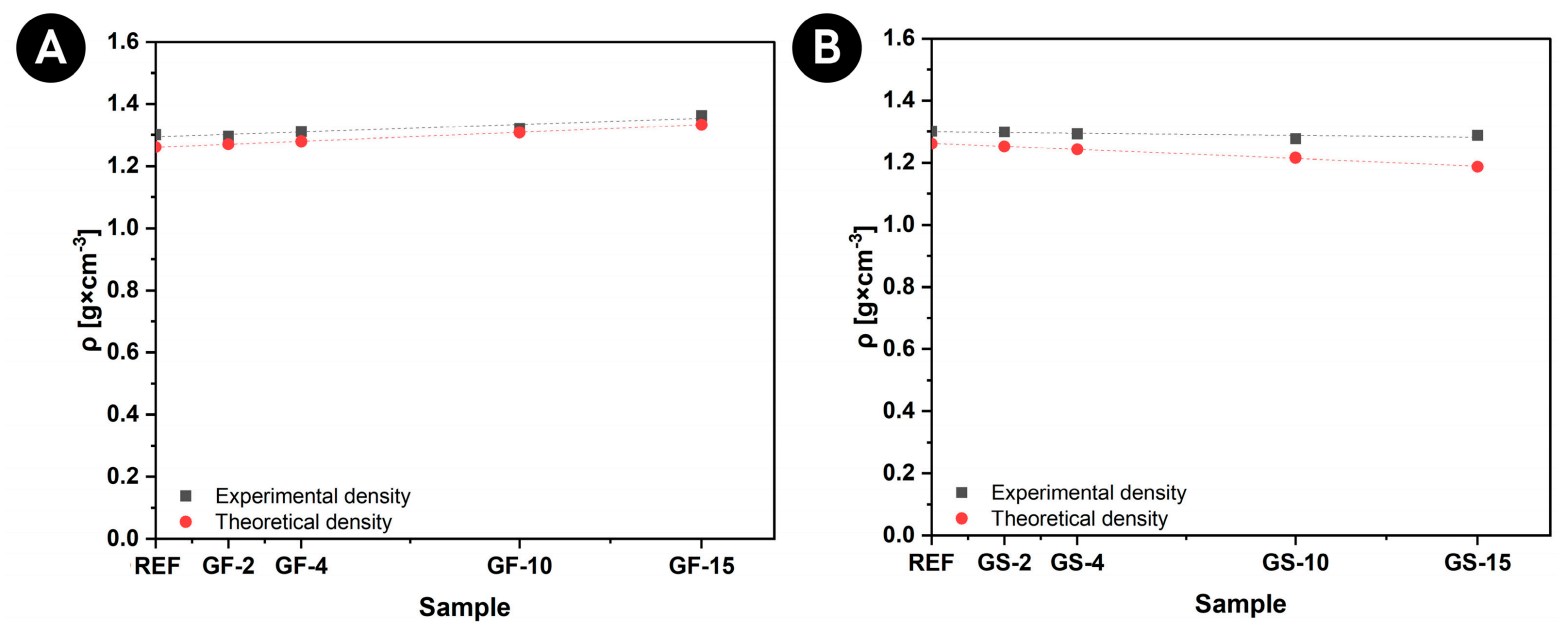
Experimental and theoretical densities of PHBV + PBAT + TPS composites containing (**A**) glass fibers and (**B**) glass spheres.

The experimental and theoretical densities of the composites are compared in Figure 10. The density of the glass-fiber-filled composites increased with increasing fiber content, whereas the density of the glass-sphere-filled composites decreased with increasing sphere content.

For the hollow-glass-sphere-filled composites, the experimental bulk density values were close to the corresponding theoretical estimates over the investigated composition range. The glass-fiber-filled composites also followed the expected composition-dependent trend, although somewhat larger differences between experimental and theoretical values were observed at higher fiber contents.

### 3.5. Tensile Properties

**Figure 11:**
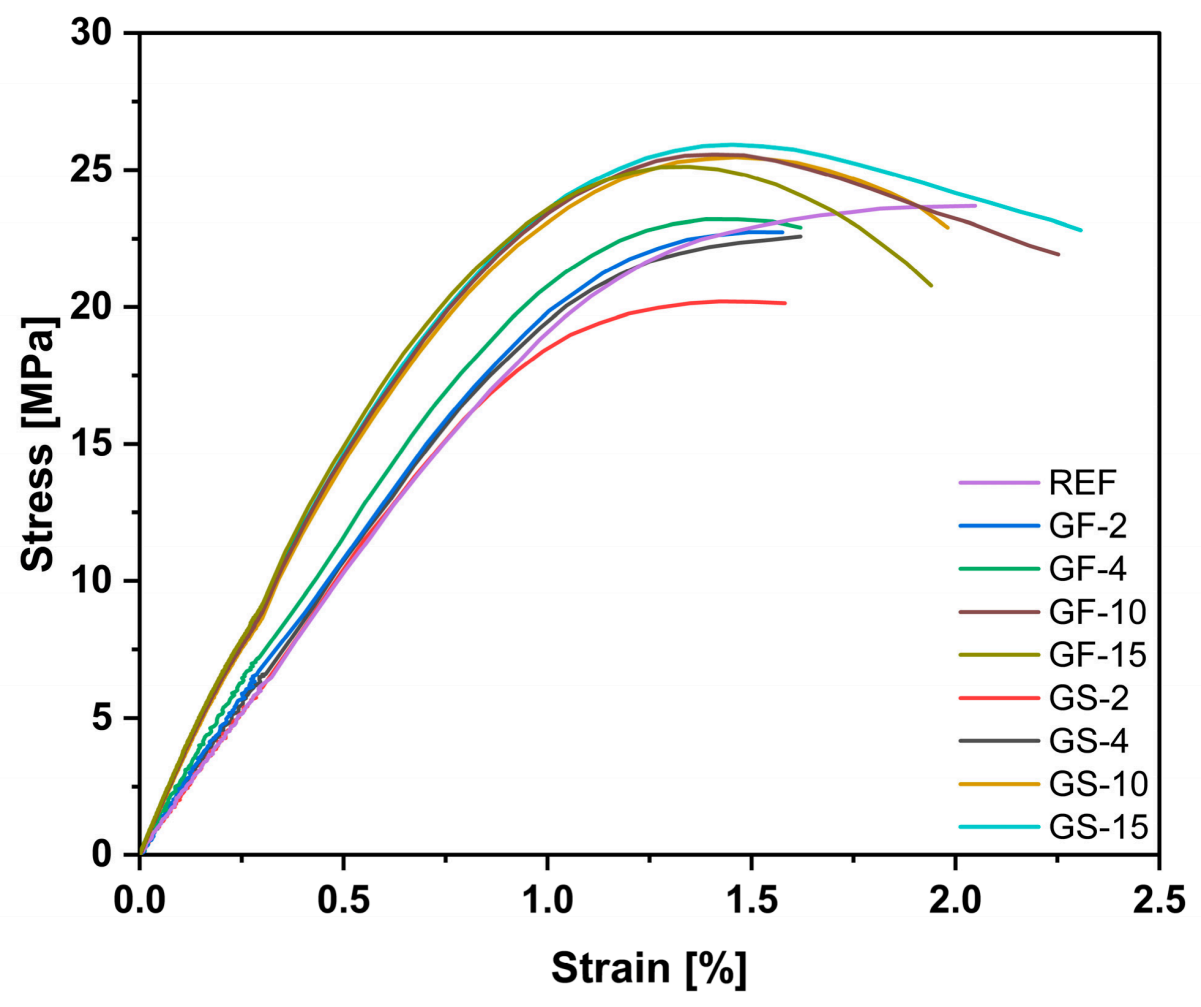
Representative tensile stress–strain curves of the unfilled PHBV + PBAT + TPS matrix and composites containing different volume fractions of glass fibers or glass spheres.

Representative tensile stress–strain curves of the unfilled PHBV + PBAT + TPS matrix and the corresponding glass-filled composites are presented in Figure 11. The tensile modulus, maximum tensile stress, stress at break, and elongation at break are summarized in Table 11. All investigated compositions exhibited relatively limited deformation before failure and similar overall stress–strain curve shapes. Clear differences were nevertheless observed depending on filler geometry and content.

The unfilled PHBV + PBAT + TPS matrix exhibited a tensile modulus of 2.06 ± 0.02 GPa, a maximum tensile stress of 23.46 ± 0.27 MPa, and an elongation at break of 2.19 ± 0.09%.

The incorporation of hollow glass spheres resulted in a non-monotonic tensile response. At sphere contents of 2.29 and 4.56 vol.%, the tensile modulus was 2.03 ± 0.03 and 2.12 ± 0.03 GPa, respectively, and remained close to that of the unfilled matrix. The corresponding maximum tensile stresses were 20.15 ± 0.23 and 22.44 ± 0.33 MPa.

At higher hollow glass-sphere contents, both the tensile modulus and maximum tensile stress decreased. At 11.31 vol.% glass spheres, the tensile modulus was 1.74 ± 0.06 GPa and the maximum tensile stress was 18.01 ± 0.20 MPa. At 16.84 vol.%, these values decreased further to 1.71 ± 0.04 GPa and 15.92 ± 0.14 MPa, respectively.

The stress at break also decreased at the two highest sphere contents, reaching 10.18 ± 0.69 MPa at 11.31 vol.% and 8.68 ± 0.23 MPa at 16.84 vol.%. The corresponding elongations at break were 7.99 ± 2.04% and 3.75 ± 0.38%. The composite containing 11.31 vol.% hollow glass spheres showed the highest elongation at break and the largest scatter among the investigated compositions.

A different trend was observed for the glass-fiber-filled composites. The tensile modulus increased progressively with increasing fiber content, from 2.28 ± 0.03 GPa at 1.02 vol.% to 2.47 ± 0.03 GPa at 2.06 vol.%, 2.92 ± 0.07 GPa at 5.31 vol.% and 3.45 ± 0.04 GPa at 8.18 vol.%.

At the two lowest fiber contents, the maximum tensile stress was 22.61 ± 0.15 and 23.12 ± 0.23 MPa, respectively. At higher fiber contents, it increased to 25.55 ± 0.45 MPa at 5.31 vol.% and 27.32 ± 0.33 MPa at 8.18 vol.%. The highest tensile modulus and maximum tensile stress among all investigated materials were therefore recorded for the composite containing 8.18 vol.% glass fibers.

The elongation at break of the fiber-filled composites remained within a relatively narrow range of 1.59–2.27%. The stress at break varied from 21.60 ± 0.66 to 24.40 ± 0.41 MPa and remained lower than or close to the corresponding maximum tensile stress.

**Figure 12:**
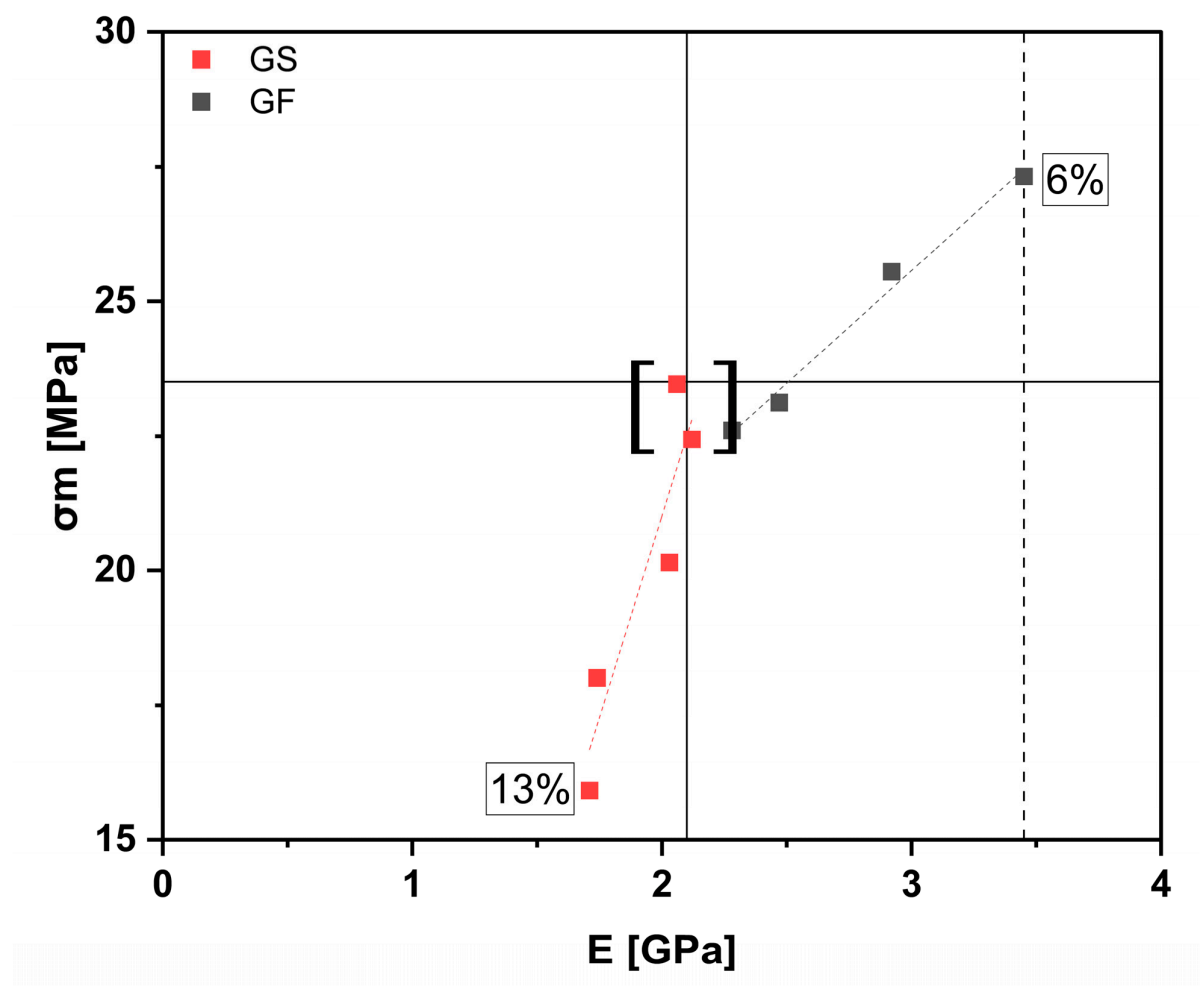
Relationship between maximum tensile stress and tensile modulus for PHBV + PBAT + TPS composites containing glass fibers or glass spheres. The reference lines indicate the properties of the unfilled PHBV + PBAT + TPS matrix.

The relationship between maximum tensile stress and tensile modulus is presented in Figure 12. Increasing glass-fiber content shifted the composites toward higher tensile modulus and, at the two highest fiber contents, higher maximum tensile stress. In contrast, at the two lowest hollow-glass-sphere contents, the tensile modulus remained close to the reference value, whereas the mean maximum tensile stress was lower. At the two highest sphere contents, both tensile modulus and maximum tensile stress were lower than those of the reference matrix.

### 3.6. Charpy Impact Strength

**Figure 13:**
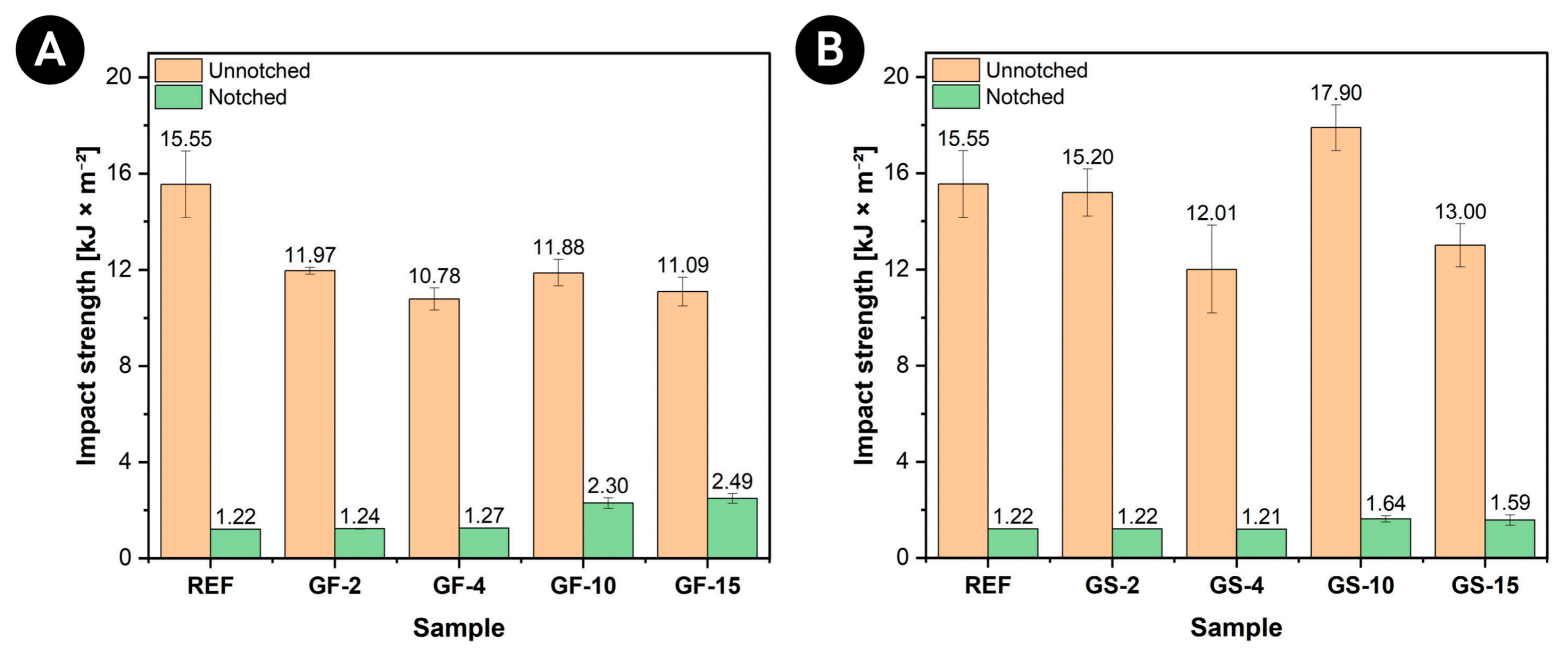
Charpy impact strength of notched and unnotched PHBV + PBAT + TPS specimens containing different volume fractions of (**A**) glass fibers and (**B**) glass spheres.

The Charpy impact strength of the unfilled PHBV + PBAT + TPS matrix and the corresponding glass-filled composites is presented in Figure 13 and summarized in Table 12. The tests were performed using both notched and unnotched specimens.

A clear difference was observed between the two specimen configurations. The notched specimens exhibited low impact-strength values for all investigated compositions. In contrast, the impact strength of all unnotched specimens remained above 10 kJ × m^−2^.

For the glass-fiber-filled composites, the unnotched impact-strength values varied within a relatively limited range. The incorporation of glass fibers did not produce a progressive increase in impact strength with increasing fiber content. The values generally remained below that of the unfilled PHBV + PBAT + TPS matrix.

The hollow-glass-sphere-filled composites exhibited a non-monotonic response in the unnotched configuration. The impact strength initially decreased with increasing sphere content, subsequently increased and reached the highest value within the sphere-filled series at 11.31 vol.% glass spheres, and then decreased again at 16.84 vol.%.

For the notched specimens, only limited composition-dependent differences were observed in both filler series. Neither glass fibers nor glass spheres produced a systematic improvement in the notched impact strength.

## 4. Discussion

### 4.1. Multiphase Matrix and Filler-Induced Crystalline Changes

The combined DSC and XRD results confirmed that the crystalline response of the PHBV + PBAT + TPS matrix was governed mainly by the PHBV phase. Neat PHBV exhibited the highest crystallinity among the investigated polymer components, whereas PBAT showed a substantially smaller crystalline contribution and TPS was predominantly amorphous. Consequently, the thermal response of the unfilled ternary blend was dominated by the PHBV melting transition, with a weaker contribution from PBAT and no distinct melting transition attributable to TPS. This behavior is consistent with the multiphase character of the matrix, in which the constituent polymers retain their individual thermal and structural characteristics rather than forming a fully homogeneous molecular system.

The preservation of the characteristic PHBV- and PBAT-related diffraction reflections in the unfilled blend further supports the coexistence of separate crystalline structures. No additional reflections indicating the formation of a new crystalline phase were detected. Therefore, the XRD results provide no evidence of detectable co-crystallization between the blend components. The absence of distinct TPS-related reflections in the blend is consistent with the low crystalline contribution of TPS and the dominance of the PHBV-related diffraction pattern.

The crystallinity values obtained by DSC and XRD differed substantially and should not be interpreted as directly interchangeable absolute measures. DSC characterizes the thermal response of the crystallizable polymer fractions during heating and depends on the selected reference enthalpies, the assumed contribution of the individual components, and possible structural reorganization during the scan. In the present multiphase system, overlapping thermal transitions of PHBV and PBAT may additionally affect peak integration. XRD, in contrast, characterizes the crystalline order present in the solid material at room temperature and is influenced by peak overlap, background subtraction, profile fitting, and preferred orientation. The DSC-based apparent crystallinity and the composite-level XRD-based crystallinity index should therefore be regarded as complementary method-dependent descriptors of the thermal and structural state of the PHBV + PBAT + TPS-based materials rather than as directly interchangeable estimates of the same absolute crystalline fraction.

The incorporation of glass fibers and glass spheres did not produce detectable new crystalline phases, but it modified the relative crystalline contribution and texture of the injection-molded composites. At the lowest hollow-glass-sphere content, the composite-level XRD-based crystallinity index increased slightly from 59.1% to 60.5%. This limited increase may be consistent with a weak heterogeneous nucleation effect of the glass surface at low particle loading. However, the available DSC and XRD results do not allow this contribution to be separated unambiguously from changes in diffraction intensity, preferred orientation, and profile fitting. The increase should therefore be interpreted cautiously.

At higher filler contents, the composite-level XRD-based crystallinity index decreased in both composite series and reached approximately 47% at the highest loadings. Because this index was calculated for the entire composite, its reduction does not necessarily represent an equivalent decrease in the intrinsic crystallinity of the polymer matrix. Glass fibers and hollow glass spheres contribute predominantly to the non-crystalline scattering background, and their increasing proportion changes the relative contribution of the polymer-related crystalline reflections to the total diffraction profile. Nevertheless, the concurrent changes in reflection intensities and texture index indicate that the fillers also affected the crystalline organization developed during injection molding.

The Scherrer-derived apparent PHBV (110) coherent-domain size remained within approximately 21–24 nm over the investigated composition range. Thus, the changes observed in the composite-level XRD-based crystallinity index were not accompanied by a systematic change in this particular PHBV coherent-domain dimension. This observation should not be interpreted as evidence that the fillers predominantly changed the amount or orientation of crystalline material, because crystallinity index, coherent-domain size, and preferred orientation are independent structural descriptors. The texture index provides separate information on changes in the preferred orientation of PHBV crystalline domains.

The texture-index results further indicate filler-dependent differences in the preferred orientation of PHBV crystalline domains developed during processing. The unfilled PHBV + PBAT + TPS blend exhibited preferred PHBV crystalline orientation, which is consistent with processing-induced organization under shear during injection molding. Hollow glass spheres caused only limited changes in the texture index at low and intermediate contents, followed by a decrease at the highest loading, whereas the fiber-filled series showed greater variation. A quantitative orientation analysis was attempted using the segmented fiber cross-sections (Section 3.4). Within the range of section geometries accessible in these micrographs, the measured orientation descriptors did not differ from those expected for an isotropic fiber population, so the present data neither establish nor exclude a preferred fiber orientation. Flow-induced alignment during injection molding remains a plausible interpretation, consistent with the elongated fiber profiles observed on the fracture surfaces and with the processing route applied, but it is not demonstrated by the present measurements, and no causal relationship between fiber orientation and the PHBV texture index is claimed.

Importantly, the differences in mechanical behavior between the two composite series cannot be explained by crystallinity alone. At higher filler contents, the composite-level XRD-based crystallinity index decreased for both glass-fiber- and hollow-glass-sphere-filled materials. Nevertheless, only the fiber-filled composites exhibited a pronounced increase in tensile modulus and maximum tensile stress. The different tensile responses of the two-filler series therefore cannot be explained by the composite-level crystalline descriptors alone. The increase in tensile modulus and maximum tensile stress in the fiber-filled composites is consistent with direct reinforcement by the high-aspect-ratio fibers. The measured sectional fiber anisotropy may also contribute to this response; however, its contribution cannot be separated quantitatively from filler content, geometry, and interfacial effects on the basis of the present dataset.

### 4.2. Geometry-Dependent Melt Flow and Microstructural State

The MFR results demonstrated that glass fibers and glass spheres modified the flow response of the same PHBV + PBAT + TPS matrix in distinctly different ways. The unfilled blend exhibited an MFR of 13.6 g × 10 min^−1^. In the fiber-filled series, the MFR decreased progressively with increasing filler content, reaching 5.9 g × 10 min^−1^ at 8.18 vol.% glass fibers. In contrast, the glass-sphere-filled composites showed a non-monotonic response: the MFR initially increased to 18.8 and 26.8 g × 10 min^−1^ at 2.29 and 4.56 vol.% glass spheres, respectively, and then decreased below the value of the unfilled matrix at higher sphere contents.

The progressive reduction in MFR observed for the glass-fiber-filled composites indicates lower apparent melt flowability with increasing contents of high-aspect-ratio inclusions. Such a trend is consistent with the generally higher resistance to melt flow associated with elongated fillers; however, the present MFR measurements do not allow the underlying rheological mechanism to be identified.

The reduction in MFR coincided with an increase in the specific external polymer–glass interfacial area from 4.35 to 29.39 mm^−1^. The accompanying changes in inter-fiber spacing are discussed below on the basis of the revised SEM stereological analysis. These observations demonstrate concurrent changes in apparent melt flowability and microstructural descriptors with increasing fiber content, without establishing a specific rheological mechanism.

The glass-sphere-filled composites exhibited two distinct concentration-dependent regimes. At low and intermediate sphere contents, the particles were comparatively widely separated, and the measured center-to-center distances remained above approximately 26 μm. Under these conditions, the spheres occurred predominantly as isolated or locally neighboring inclusions and did not produce the same progressive flow restriction as the fibers. The increase in MFR at 2.29 and 4.56 vol.% glass spheres should not, however, be interpreted as direct evidence of a lubricating effect. MFR is a single-point measurement and does not provide information on complex viscosity, viscoelastic moduli, yield behavior, or shear-thinning over a range of shear rates. The observed increase may therefore reflect a combination of changes in phase arrangement, local shear conditions, and filler–matrix interactions, which cannot be separated using the present dataset.

At higher hollow-glass-sphere contents, the specific external polymer–glass interfacial area increased strongly, reaching 75.20 mm^−1^ at 11.31 vol.% and 107.23 mm^−1^ at 16.84 vol.%. The decrease in MFR at the higher sphere contents therefore coincided with a pronounced increase in the geometrically available external polymer–glass interfacial area. The corresponding nearest-neighbor spacing descriptors are discussed on the basis of the revised SEM stereological analysis.

The large interfacial area generated by the glass spheres resulted from their comparatively high number density and surface-to-volume relationship within the investigated composition range. The decrease in MFR at higher hollow-glass-sphere contents coincided with a substantially larger specific external polymer–glass interfacial area and changes in nearest-neighbor spacing. These concurrent changes are consistent with an increasing geometric influence of the dispersed particles on the flowing multiphase matrix; however, the underlying rheological mechanism cannot be established from the MFR measurements alone.

The two-dimensional nearest-neighbor analysis provided additional information on filler arrangement within the analyzed SEM fields. For hollow glass spheres, R ranged from 0.94 to 1.04, remaining close to the value of unity expected for a random planar arrangement at the same areal number density. For glass fibers, R ranged from 1.06 to 1.16; the slightly supra-unity values may reflect mutual exclusion associated with the elongated geometry of sectioned fibers. Together with the qualitative SEM observations, these results indicate no pronounced filler-rich clustering within the analyzed two-dimensional fields. However, the present analysis does not establish a globally random three-dimensional filler distribution throughout the molded specimens.

The experimental density values generally followed the composition-dependent trends predicted from the constituent densities. For the hollow-glass-sphere-filled composites, the measured values were close to the corresponding theoretical estimates, whereas the glass-fiber-filled composites also showed the expected increase in density with filler content, with somewhat larger deviations at higher loadings. These results indicate consistency between the measured bulk density and the calculated compositional trend. However, helium pycnometry does not provide spatial information on defects and therefore cannot exclude localized interfacial voids, damage to individual hollow glass spheres, or other small-scale processing-related defects. Accordingly, the density data were not used to assign a specific mechanism to the observed MFR behavior.

Overall, the two filler geometries produced distinctly different concentration-dependent MFR patterns. Glass fibers showed progressively lower apparent melt flowability with increasing filler content, whereas hollow glass spheres showed higher apparent flowability at low-to-intermediate contents, followed by lower apparent flowability at higher contents. These trends coincided with geometry-dependent changes in filler spacing and specific external polymer–glass interfacial area. Thus, the present results demonstrate associations among filler geometry, microstructural descriptors, and apparent melt flowability, without establishing a specific rheological mechanism.

Because only MFR measurements were performed, the proposed mechanisms should be considered interpretations of the observed comparative flow behavior rather than a complete rheological description. Oscillatory and steady-shear measurements would be required to determine how filler geometry affects viscosity, shear-thinning, elastic response and possible network formation over a broader deformation range.

### 4.3. Tensile Reinforcement and Interfacial Constraints

The tensile results showed distinctly different concentration-dependent responses for the two filler geometries, indicating that filler content alone was insufficient to describe the mechanical behavior. Although both filler series exhibited a reduction in the composite-level XRD-based crystallinity index at higher loadings, their tensile responses were opposite. Glass fibers progressively increased the tensile modulus and, at higher contents, the maximum tensile stress, whereas hollow glass spheres reduced both properties when their concentration exceeded 4.56 vol.%. Therefore, the observed tensile behavior cannot be explained solely by changes in the crystalline organization of the PHBV + PBAT + TPS matrix.

In the glass-fiber-filled composites, the tensile modulus increased from 2.06 GPa for the unfilled matrix to 3.45 GPa at 8.18 vol.% fibers, while the maximum tensile stress increased from 23.46 to 27.32 MPa. This response is consistent with the reinforcing contribution expected for high-aspect-ratio inclusions. Stress transfer through the fiber–matrix interface represents a plausible contribution to this behavior; however, interfacial adhesion and interfacial shear strength were not measured directly. A quantitative orientation analysis of the segmented fiber cross-sections did not resolve a preferred orientation within the accessible range of section geometries (Section 3.4). Flow-induced fiber alignment during injection molding therefore remains a plausible contributor to the tensile response, consistent with the anisotropic filler geometry and the processing route, rather than a measured quantity. Because the analysis does not reconstruct the three-dimensional orientation state relative to the tensile-loading direction, the magnitude of any such contribution cannot be determined from the present data.

The increase in tensile performance occurred concurrently with an increase in the specific external polymer–glass interfacial area from 4.35 to 29.39 mm^−1^. This association is consistent with an increasing contribution of the fibrous reinforcement; however, the role of the interface cannot be separated quantitatively from filler content, high-aspect-ratio geometry, and possible orientation effects. Because direct interfacial adhesion or interfacial shear-strength measurements were not performed, the increase in external interfacial area should not itself be interpreted as direct evidence of improved load transfer.

At the two lowest fiber contents, the tensile modulus increased, whereas the maximum tensile stress remained close to that of the unfilled blend. At higher fiber contents, both tensile modulus and maximum tensile stress increased. This concentration-dependent response is consistent with an increasing mechanical contribution of the fibrous reinforcement; however, the present experiments do not establish whether a reinforcement network developed or quantify the corresponding load-transfer mechanism.

At 2.29 and 4.56 vol.% hollow glass spheres, the tensile modulus remained broadly comparable to that of the reference matrix. The corresponding mean maximum tensile stresses were lower than that of the reference, particularly for the 2.29 vol.% composition. These comparisons are descriptive. At 11.31 and 16.84 vol.%, both stiffness and strength decreased markedly. Unlike fibers, spherical inclusions do not provide the same direction-dependent reinforcing geometry. Their mechanical contribution may therefore depend more strongly on local stress distribution, particle–matrix interactions, and the continuity of the polymer-rich regions between neighboring particles.

The deterioration in tensile properties at higher sphere contents coincided with an increase in the specific external polymer–glass interfacial area to 75.20 mm^−1^ at 11.31 vol.% and 107.23 mm^−1^ at 16.84 vol.%. Together with the reduced local particle spacing observed in the revised SEM analysis, these microstructural changes may favor local stress concentration or interface-related damage. However, such mechanisms were not directly verified in the present study.

The measured bulk densities of the hollow-glass-sphere-filled composites generally followed the theoretical compositional trend. However, helium pycnometry cannot exclude localized interfacial voids or damage to individual hollow particles. The concurrent decrease in tensile properties, reduction in particle spacing, and increase in specific external interfacial area are consistent with possible interface-related stress concentration or damage processes, but the occurrence and relative importance of these mechanisms were not directly determined. Possible mechanisms include local debonding, microvoid formation, and stress concentration around the spherical inclusions, as reported for other glass-particle-filled polymer systems [14,15,16,17]. Nevertheless, these mechanisms were not directly verified, because tensile fracture surfaces and interfacial adhesion were not examined.

The increased apparent elongation at break observed for the composites containing 11.31 and 16.84 vol.% hollow glass spheres should also be interpreted cautiously. In both cases, the stress at break was substantially lower than the maximum tensile stress, and the 11.31 vol.% composition exhibited considerable scatter. The higher measured elongation therefore does not necessarily indicate uniform ductilization of the matrix. It may instead be associated with a variable post-maximum damage process, during which localized interfacial separation and progressive loss of load-bearing continuity could allow additional deformation before complete specimen failure.

Overall, the tensile results show that a larger specific external polymer–glass interfacial area did not correspond uniformly to improved mechanical performance. In the glass-fiber-filled series, increasing fiber content and external interfacial area coincided with higher tensile modulus and, at higher contents, higher maximum tensile stress. In contrast, the hollow-glass-sphere-filled series exhibited a substantially larger external interfacial area, while tensile modulus and strength decreased at higher particle contents. These contrasting trends indicate that interfacial area alone is not sufficient to predict tensile response. Filler geometry, orientation, particle spacing, and interfacial interactions are plausible contributing factors, but their individual causal contributions cannot be separated quantitatively from the present dataset.

### 4.4. Impact Behavior and Loading-Mode Dependence

The Charpy results demonstrated that the effect of glass fillers depended strongly on specimen configuration and loading mode. All notched specimens exhibited low impact-strength values, whereas the unnotched compositions retained impact strengths above 10 kJ × m^−2^. This marked difference confirms the high notch sensitivity of the PHBV + PBAT + TPS-based materials and indicates that the presence of a predefined stress concentrator strongly influenced crack initiation.

In the notched specimens, fracture was initiated preferentially at the notch tip, where the local stress concentration was substantially higher than in the surrounding material. Under these conditions, the ability of the fillers to modify crack initiation was limited because the location of failure initiation was imposed geometrically. Consequently, neither glass fibers nor hollow glass spheres produced a systematic improvement in notched impact strength. The relatively small composition-dependent differences observed in this configuration suggest that the response was governed predominantly by the semi-brittle character of the matrix and by rapid crack propagation from the notch.

The unnotched specimens were more sensitive to changes in filler geometry and microstructural arrangement. In the glass-fiber-filled series, the impact-strength values remained above 10 kJ × m^−2^ but did not increase progressively with fiber content. This behavior differs from the tensile response, in which glass fibers produced clear increases in modulus and maximum tensile stress. Thus, the tensile reinforcement observed under quasi-static loading did not translate into a corresponding improvement in resistance to dynamic fracture.

Under impact loading, the increased stiffness of the composite and the limited deformation capacity of the PHBV + PBAT + TPS matrix may have influenced the amount of energy dissipated before fracture. The observed impact response may be consistent with several competing processes commonly considered for fiber-filled polymer composites, including crack bridging, fiber pull-out, interface-related damage, and changes in matrix deformation. However, because the post-impact fracture surfaces were not examined, the occurrence and relative contributions of these processes cannot be established from the present results.

The glass-sphere-filled composites exhibited a more pronounced non-monotonic response in the unnotched configuration. The impact strength initially decreased with increasing sphere content, reached the highest value within the sphere-filled series at 11.31 vol.%, and subsequently decreased again at 16.84 vol.%. This behavior contrasts with the progressive deterioration in tensile properties observed at higher sphere contents and indicates that the role of spherical inclusions differed between quasi-static and dynamic loading.

The segmentation-derived microstructural descriptors provide a basis for interpreting this concentration-dependent response. Increasing sphere content progressively reduced the edge-to-edge distance between neighboring particles and strongly increased the specific polymer–glass interfacial area. Closely spaced rigid particles can increase local stress concentrations and facilitate damage initiation. In unnotched specimens, several interface-related energy-dissipation mechanisms, including local debonding, microcracking, crack-path deflection, and localized matrix deformation, have been reported for polymer composites containing glass microspheres or other spherical glass inclusions [38,39,40,41,42]. Such mechanisms may provide a plausible interpretation of the present non-monotonic impact response, although they were not directly observed in this study.

The elevated unnotched impact strength observed at the intermediate (11.31 vol.%) hollow-glass-sphere content coincided with a particular combination of particle spacing and specific external polymer–glass interfacial area. In view of the mechanisms reported for spherical-particle-filled polymers, this response may be consistent with an increased contribution of interface-related energy-dissipation processes. However, the present measurements do not establish which fracture processes were active or whether their relative contribution changed with sphere content.

At the highest hollow-glass-sphere content (16.84 vol.%), the further increase in specific external polymer–glass interfacial area to 107.23 mm^−1^ coincided with a reduction in unnotched impact strength. Earlier interaction between local damage zones represents one possible interpretation of this trend; however, this mechanism was not verified experimentally.

The opposite tensile and impact trends of the sphere-filled composites illustrate that filler performance cannot be evaluated independently of loading conditions. Under tensile loading, the increasing number of closely spaced sphere–matrix interfaces coincided with reduced load-bearing continuity and lower stiffness and strength. Under unnotched impact loading, the non-monotonic response at intermediate sphere content may be consistent with a contribution from interface-related energy-dissipation processes; however, the present data do not establish a causal relationship between these mechanisms and the measured impact strength.

However, the proposed mechanisms were not directly verified in the present study. No post-impact fractographic analysis was performed, and particle debonding, crack deflection, microcracking, and damage-zone interaction were not quantified. These mechanisms should therefore be regarded as literature-supported interpretations of the observed trends rather than as fracture processes directly demonstrated by the experimental results. Future post-impact SEM examination of the fracture surfaces would therefore be required to verify and distinguish the relative contributions of interfacial debonding, matrix deformation, fiber pull-out, microcracking, and crack-path modification.

### 4.5. Integrated Structure–Processing–Property Relationships and Study Limitations

The results of this study reveal distinct relationships among filler geometry, structural descriptors, apparent melt flowability, and mechanical response within the same PHBV + PBAT + TPS matrix. The two glass fillers were introduced into the same polymer matrix using the same processing route and temperature program, while selected extrusion parameters varied within the reported ranges to maintain stable processing. This controlled experimental framework enabled the geometry-dependent responses of the two-filler series to be compared without changing the nominal polymer-matrix composition.

The hollow-glass-sphere-filled composites exhibited a different concentration-dependent response. At low contents, higher MFR values were observed without a corresponding improvement in tensile properties. At higher contents, decreasing particle spacing and increasing specific external polymer–glass interfacial area coincided with lower MFR, tensile modulus, and tensile strength. These associations show that a larger external interfacial area does not necessarily correspond to improved tensile performance. Possible contributions from local stress concentration, interfacial damage, and changes in the continuity of polymer-rich regions remain mechanistic interpretations because they were not measured directly.

The comparison between the two-filler series therefore shows that specific external interfacial area should not be interpreted independently of filler geometry. Similar changes in interfacial area can accompany different mechanical responses depending on the morphology and spatial arrangement of the reinforcement. In the present dataset, the larger interfacial area of the hollow-glass-sphere-filled composites did not correspond to stronger tensile reinforcement than that observed for the fiber-filled series.

The mechanical response was also strongly dependent on loading mode. The high-aspect-ratio fibers improved tensile stiffness and strength but did not produce a corresponding increase in Charpy impact strength. Conversely, hollow glass spheres reduced tensile properties at higher contents but generated a non-monotonic unnotched impact response. This difference shows that the relationship between filler microstructure and mechanical response depends on loading mode. The non-monotonic impact behavior may be consistent with additional localized energy-dissipation processes under dynamic loading, but these processes were not directly identified in the present study. Filler efficiency should therefore be evaluated in relation to the intended loading conditions rather than on the basis of a single mechanical parameter.

The results also highlight the importance of distinguishing composite-level crystallinity from direct reinforcement effects. Both filler series showed a reduction in the XRD-based crystallinity index at higher loadings, while their tensile responses differed substantially. Therefore, the differences in mechanical behavior cannot be explained by the composite-level crystalline descriptors alone. They were observed concurrently with differences in filler aspect ratio, sectional orientation, spacing, and specific external interfacial area; however, the individual contributions of these variables and of polymer–glass interfacial adhesion were not resolved quantitatively.

Several limitations should be considered when interpreting the results. A central limitation concerns the level of experimental replication. Each formulation was produced as a single compounding batch. Consequently, the specimen-to-specimen variability determined from the tensile and Charpy tests represents within-batch variability and does not quantify batch-to-batch processing reproducibility. Similarly, repeated MFR and density determinations represent technical measurement repeatability, whereas DSC and XRD were performed once per formulation, and the SEM fields constitute spatial subsampling within one molded specimen. The formulation-dependent trends should therefore be interpreted as comparative results for the investigated processing campaign rather than as population-level estimates incorporating independent batch-to-batch variability.

First, the moisture content of the individual polymer components was not independently determined immediately before melt compounding. Although vacuum degassing was used during extrusion and the compounded pellets were subsequently dried before injection molding, variability in the initial moisture condition of the raw materials, particularly TPS, cannot be completely excluded.

Second, melt behavior was evaluated using MFR measurements performed at a single temperature and load. These measurements provide a comparative indicator of apparent flowability but do not describe complex viscosity, viscoelastic response, shear-thinning behavior, or the possible formation of filler networks. In addition, agreement between experimental and theoretical bulk density does not exclude localized interfacial voids or damage to individual hollow glass spheres, because helium pycnometry does not resolve the spatial origin of such defects.

Third, the microstructural analysis was based on two-dimensional SEM sections. For elongated glass fibers, the measured spacing and dispersion descriptors were influenced by fiber orientation, sectioning angle, and intersection of the fibers with the fracture plane. The obtained stereological parameters should therefore be treated as comparative descriptors of the analyzed two-dimensional fields rather than as evidence of a globally random or complete three-dimensional filler distribution throughout the molded specimens.

Fourth, the deep-learning segmentation models were trained using a limited number of SEM images. Transfer learning and data augmentation improved the robustness of the analysis; however, validation showed different detection performance for the two reinforcement classes. The glass-fiber detector achieved a count recall of 97.1%, whereas the hollow-glass-sphere detector achieved 70.3%. Consequently, segmentation-derived descriptors for the hollow glass spheres, particularly the measured particle-size distribution, may be biased toward larger and more readily detected particles. The reported descriptors therefore characterize the segmented objects within the analyzed SEM fields rather than the complete particle population of the bulk molded material. In addition, the models were not intended to quantify sub-resolution interfacial defects, nanoscale pores, or local chemical differences between the polymer phases.

Fifth, no direct interfacial adhesion or interfacial shear-strength measurements were performed. Consequently, the proposed contribution of polymer–glass load transfer cannot be quantified directly. Fiber orientation was quantified from the segmented cross-sections, as described in Section 3.4; however, this analysis characterizes the out-of-plane orientation relative to the analyzed sections and does not resolve the complete three-dimensional fiber-orientation state relative to the tensile-loading direction.

Finally, post-tensile and post-impact fractographic analyses were not conducted. The proposed mechanisms involving particle–matrix debonding, microvoid formation, fiber pull-out, crack bridging, microcracking, and crack-path deflection should therefore be regarded as literature-supported interpretations. Future SEM examination of post-tensile and post-impact fracture surfaces would be required to verify and distinguish their relative contributions.

Despite these limitations, the combined DSC, XRD, MFR, density, SEM segmentation, and mechanical results provide a consistent comparison of two geometrically distinct glass fillers in the same multiphase PHBV + PBAT + TPS matrix. The study demonstrates that filler selection should account simultaneously for processing response, crystalline organization, filler spacing, interfacial area, and loading mode. Glass fibers were more effective when increased stiffness and tensile strength were required, whereas hollow glass spheres produced a more complex concentration-dependent response that may be relevant where flow behavior and unnotched impact performance must be balanced.

## 5. Conclusions

This study demonstrated distinct geometry-dependent structural, processing, and mechanical responses in composites based on the same multiphase PHBV + PBAT + TPS matrix. The use of an identically processed unfilled blend as the reference enabled the effects of glass fibers and hollow glass spheres to be distinguished from the intrinsic behavior of the ternary polymer matrix.

The thermal and crystalline response of the investigated materials was dominated by PHBV. Neither glass fibers nor hollow glass spheres induced detectable new crystalline phases. At higher filler contents, the composite-level XRD-based crystallinity index decreased to approximately 47%, whereas the Scherrer-derived apparent PHBV (110) coherent-domain size remained within approximately 21–24 nm. The texture index varied independently with filler type and content. These descriptors characterize different aspects of the crystalline structure and should therefore not be interpreted as mutually interchangeable measures of crystallinity.

Within the analyzed two-dimensional SEM fields, no pronounced filler-rich clustering was evident, while the quantitative stereological descriptors revealed distinct geometry-dependent differences in local filler arrangement and specific external polymer–glass interfacial area. For the hollow-glass-sphere-filled composites, the specific external interfacial area increased to 107.23 mm^−1^ at 16.84 vol.%, whereas for the glass-fiber-filled composites, it reached 29.39 mm^−1^ at 8.18 vol.%. These values demonstrate the substantially larger geometrically available external polymer–glass interface generated by the hollow spherical filler over the investigated composition range.

The two filler geometries also produced different melt-flow and tensile responses. Glass fibers progressively reduced MFR and exhibited increasing tensile modulus and, at higher contents, increasing maximum tensile stress. At 8.18 vol.% fibers, the tensile modulus increased from 2.06 to 3.45 GPa, and the maximum tensile stress increased from 23.46 to 27.32 MPa. Hollow glass spheres produced a non-monotonic MFR response and, at higher contents, reduced tensile modulus and strength. In the sphere-filled series, the reductions in tensile properties at higher contents coincided with decreasing interparticle spacing and increasing specific external polymer–glass interfacial area. These results show that external interfacial area alone is not sufficient to predict tensile performance.

The impact behavior depended strongly on specimen configuration and loading mode. All notched specimens exhibited low impact strength, whereas the unnotched compositions retained values above 10 kJ × m^−2^. Glass fibers did not provide the progressive improvement in impact strength observed under tensile loading. Hollow glass spheres exhibited a non-monotonic unnotched impact response, with the highest value within the sphere-filled series obtained at 11.31 vol.%. This response differed from the tensile trend, emphasizing the loading-mode dependence of the measured mechanical behavior.

Overall, high-aspect-ratio glass fibers were more effective in increasing stiffness and tensile strength, whereas hollow glass spheres produced a more complex response controlled by their content, spacing, interfacial area, and loading conditions. The results demonstrate that the selection of glass reinforcement for PHBV + PBAT + TPS-based composites should account simultaneously for filler geometry, melt flowability, crystalline organization, microstructural arrangement, and the intended mode of mechanical loading. Within the investigated property range, the fiber-filled materials appear particularly relevant for low-loaded components requiring enhanced stiffness, whereas the sphere-filled systems may be considered where a balance between processing behavior and impact response is of interest; application-specific validation would nevertheless be required. The present results concern the structural, processing, and mechanical behavior of glass-filled composites based on a biodegradable polymer matrix; biodegradation of the complete composites was not investigated.

## Figures and Tables

**Table 1:** Selected properties of PHBV, PBAT, and TPS.

Property	PHBV	PBAT	TPS
Density [g × cm^−3^]	1.25	1.21	1.35
Yield stress [MPa]	31–36	n/a	22
Tensile strength [MPa]	38	≥25	20
Elongation at break [%]	3.9	n/a	2
Young’s modulus [GPa]	2.80–3.50	n/a	1.10
Flexural modulus [GPa]	3.52–4.17	n/a	n/a
MFR, 190 °C, 2.16 kg [g × 10 min^−1^]	15.6	2.5–4.5	20
Vicat softening temperature [°C]	166	≥80	61
DSC melting point [°C]	170–176	116–122	amorphous

**Table 2:** Properties of reinforcement materials used in manufactured composites.

Reinforcement Material	Dimensions	Density [g × cm^−3^]
Glass fibers	10 μm of diameter 4.5 mm of length	2.5
Hollow glass spheres	9–13 μm of external diameter	1.1 *

* *Supplier-reported density used as the effective density of the complete hollow particle*.

**Table 3:** Temperature profile.

Zone	I (Backfill)	II-X	XI	Head	Melt Temp.	P [MPa]
[°C]	35	150	160	160	167–170	4.2

**Table 4:** Designation and composition of the tested composites.

Sample Code	Sample	Hollow Glass Spheres		Glass Fibers	
		wt.%	vol.%	wt.%	vol.%
REF	unfilled PHBV + PBAT + TPS	0	0	0	0
GS-2	PHBV + PBAT + TPS + 2 wt.%GlSph	2	2.29	0	0
GS-4	PHBV + PBAT + TPS + 4 wt.%GlSph	4	4.56	0	0
GS-10	PHBV + PBAT + TPS + 10 wt.%GlSph	10	11.31	0	0
GS-15	PHBV + PBAT + TPS + 15 wt.%GlSph	15	16.84	0	0
GF-2	PHBV + PBAT + TPS + 2 wt.%GlFib	0	0	2	1.02
GF-4	PHBV + PBAT + TPS + 4 wt.%GlFib	0	0	4	2.06
GF-10	PHBV + PBAT + TPS + 10 wt.%GlFib	0	0	10	5.31
GF-15	PHBV + PBAT + TPS + 15 wt.%GlFib	0	0	15	8.18

**Table 5:** COCO instance-segmentation mask metrics for the glass-fiber and hollow-glass-sphere detectors computed on the held-out validation sets at a score threshold of 0.05. APL was undefined because no annotated object reached the COCO large-object threshold.

Detector	Validation Images	Annotated Instances	AP	AP50	AP75	APS	AR@100	Count Recall
Glass fibers	8	34	32.8	46.9	44.9	30.9	42.7	97.1%
Hollow glass spheres	8	37	26.2	45.9	24.7	25.2	30.3	70.3%

**Table 6:** Melting enthalpies and DSC-based crystallinity values of the neat polymer components and the unfilled PHBV + PBAT + TPS blend.

	ΔH_exp_ [J × g^−1^]	ΔH_ref_ [J × g^−1^]	$X_{C}^{DSC}$[%]
PHBV	98.2	147	66.8
PBAT	9.2	114	8.07
TPS	Amorphous	Amorphous	Amorphous
PHBV + PBAT + TPS	33.4	–	32.75

**Table 7:** Crystalline structure parameters and XRD-based crystallinity indices of the neat polymer components and the unfilled PHBV + PBAT + TPS blend.

Material		Crystal System	Calculated Lattice Parameters [Å]	XRD-Based Crystallinity Index, $X_{c}^{XRD}$[%]
PHBV		orthorhombic ($P2_{1}2_{1}2_{1}$)	a = 5.70 ± 0.001	77.4
			b = 13.13 ± 0.003	
			c = 5.94 ± 0.002	
PBAT *		triclinic ($P\bar{1}$)	a = 4.85 ± 0.03	37.5
			b = 5.89 ± 0.03	
			c = 12.54 ± 0.44	
TPS	B-type	hexagonal ($P6_{1})$	N.A.	11.3
	V-type (hydrated)			
	V-type (anhydrous)			
PHBV+ PBAT + TPS	PHBV	orthorhombic ($P2_{1}2_{1}2_{1}$)	a = 5.67 ± 0.09	59.1
			b = 13.23 ± 0.10	
			c = 5.94 ± 0.14	
	PBAT	triclinic ($P\bar{1}$)	a = 5.05 ± 0.49	
			b = 5.98 ± 0.17	
			c = 10.26 ± 2.75	

* *Lattice angles fixed to α = 99.9°; β = 115.2°; γ = 111.3° based on PBT structure* [37].

**Table 8:** Melt mass-flow rate of the unfilled PHBV + PBAT + TPS matrix and composites containing different volume fractions of glass spheres or glass fibers.

Sample	MFR [g × 10 min^−1^]
REF	13.6
GS-2	18.8
GS-4	26.8
GS-10	8.5
GS-15	7.6
GF-2	12.6
GF-4	11.7
GF-10	6.5
GF-15	5.9

**Table 9:** Segmentation-derived stereological descriptors of PHBV + PBAT + TPS composites containing glass fibers or glass spheres.

Sample	vol.%	Fields	*n*	*D* [µm]	*C2C_measured_* [µm]	E2E [µm]	C2C_random2D_ [µm]	*S_V_* [mm^−1^]	*R*
GF-2	1.02	4	60	9.37	74.8 ± 20.8	65.5 ± 20.8	63.6	4.35	1.16 ± 0.18
GF-4	2.06	5	92	9.99	60.5 ± 12.0	50.5 ± 11.9	56.5	8.25	1.07 ± 0.20
GF-10	5.31	5	131	11.74	50.2 ± 6.4	38.5 ± 6.4	47.3	18.09	1.06 ± 0.06
GF-15	8.18	5	136	11.13	49.3 ± 7.7	38.1 ± 7.4	46.4	29.39	1.06 ± 0.10
GS-2	2.29	3	58	10.99	55.7 ± 6.7	44.7 ± 7.0	56.0	12.49	1.00 ± 0.12
GS-4	4.56	5	44	11.60	77.2 ± 6.6	65.5 ± 8.0	82.4	23.60	0.95 ± 0.17
GS-10	11.31	5	82	9.02	56.3 ± 6.0	47.3 ± 6.4	59.8	75.20	0.94 ± 0.10
GS-15	16.84	5	194	9.42	40.1 ± 3.5	30.7 ± 3.5	38.7	107.23	1.04 ± 0.06

**Table 10:** Size-distribution statistics of hollow glass spheres and glass fibers determined from deep-learning-segmented SEM images. Equivalent external diameter is reported for hollow glass spheres, whereas the characteristic fiber diameter corresponds to the minor axis of the best-fit ellipse. Dimensions are reported in µm; “Fields” denotes the number of analyzed SEM fields, and n is the total number of segmented objects.

Sample	Filler vol.%	Fields	n	Mean	SD	Median/D50	Min	Max	D10	D90
GS-2	2.29	3	58	10.99	3.61	10.56	6.10	25.67	7.23	13.72
GS-4	4.56	5	44	11.60	4.89	10.24	5.80	28.61	6.96	17.28
GS-10	11.31	5	82	9.02	3.20	8.32	5.30	24.38	6.16	12.31
GS-15	16.84	5	194	9.42	2.32	8.84	5.23	17.08	6.77	12.86
GF-2	1.02	4	60	9.37	4.26	8.40	3.60	24.17	5.67	15.52
GF-4	2.06	5	92	9.99	3.84	8.98	4.97	24.09	6.20	15.35
GF-10	5.31	5	131	11.74	4.72	10.81	3.45	28.50	6.85	17.69
GF-15	8.18	5	136	11.13	3.62	10.41	5.77	28.60	7.24	14.85

**Table 11:** Tensile properties of the unfilled PHBV + PBAT + TPS matrix and composites containing different volume fractions of glass spheres or glass fibers. Values are presented as mean ± standard deviation.

Sample	E [GPa]	σ_m_ [MPa]	σ_b_ [MPa]	A [%]
REF	2.06 ± 0.02	23.46 ± 0.27	23.30 ± 0.25	2.19 ± 0.09
GS-2	2.03 ± 0.03	20.15 ± 0.23	19.86 ± 0.31	1.71 ± 0.12
GS-4	2.12 ± 0.03	22.44 ± 0.33	22.32 ± 0.23	1.83 ± 0.19
GS-10	1.74 ± 0.06	18.01 ± 0.20	10.18 ± 0.69	7.99 ± 2.04
GS-15	1.71 ± 0.04	15.92 ± 0.14	8.68 ± 0.23	3.75 ± 0.38
GF-2	2.28 ± 0.03	22.61 ± 0.15	22.42 ± 0.22	1.68 ± 0.06
GF-4	2.47 ± 0.03	23.12 ± 0.23	22.93 ± 0.22	1.59 ± 0.02
GF-10	2.92 ± 0.07	25.55 ± 0.45	21.60 ± 0.66	2.27 ± 0.22
GF-15	3.45 ± 0.04	27.32 ± 0.33	24.4 ± 0.41	1.72 ± 0.02

**Table 12:** Charpy impact strength of notched and unnotched PHBV + PBAT + TPS specimens containing different volume fractions of glass spheres (GSs) and fibers (GFs). Values are presented as mean ± standard deviation (n = 4) for each specimen configuration and formulation.

Sample	Unnotched [kJ × m^−2^]	Notched [kJ × m^−2^]
REF	15.55 ± 1.40	1.22 ± 0.01
GS-2	15.20 ± 0.98	1.22 ± 0.01
GS-4	12.01 ± 1.82	1.21 ± 0.01
GS-10	17.90 ± 0.95	1.64 ± 0.13
GS-15	13.00 ± 0.89	1.59 ± 0.21
GF-2	11.97 ± 0.14	1.24 ± 0.02
GF-4	10.78 ± 0.46	1.27 ± 0.01
GF-10	11.88 ± 0.55	2.30 ± 0.22
GF-15	11.09 ± 0.61	2.49 ± 0.20

## Data Availability

The data supporting the results of this study, including the trained Cascade Mask R-CNN and Mask R-CNN model weights, their Detectron2 configuration files, COCO-format manual annotations, SEM micrographs used for quantitative analysis, and per-field stereological measurements underlying the reported quantitative descriptors, are available from the corresponding author upon reasonable request. The model weight files are not publicly archived because of their size. All other data supporting the reported results are contained within the article and its Supplementary Materials.

## Supplementary Materials

The following supporting information can be downloaded at https://www.mdpi.com/article/10.3390/polym18182233/s1, Figure S1: Photo of specimens for investigations of microstructure and properties of the composites; Figure S2: Representative SEM image-processing workflow used for deep-learning-based segmentation. The figure shows examples of raw SEM micrographs, corresponding manual annotations, and segmentation overlays used to identify glass fillers in the PHBV + PBAT + TPS matrix. Panels (A–C) present SEM images of glass spheres, with the spheres highlighted in green, whereas panels (D–F) show glass fibers, highlighted in red. These data illustrate the procedure applied to extract quantitative stereological parameters, including filler spacing, nearest-neighbor index (R), and polymer–glass interfacial area. Table S1: Manual versus automatic size measurements on the independent validation images. Instances were matched one-to-one at a mask IoU ≥ 0.50. Bias denotes automatic-manual and MAD denotes mean absolute deviation; Table S2: Complete training and inference configuration of the glass-fiber and hollow-glass-sphere detectors; Table S3: Multi-peak Voigt deconvolution goodness-of-fit statistics ($R^{2}$ and $\chi_{v}^{2}$), flow-induced planar texture indices ($A_{020}/A_{110}$), and sensitivity comparison between uncorrected ($X_{c}^{raw}$) and ICDD-corrected ($X_{c}^{corr}$) XRD crystallinity indices, alongside discrepancies between those values ($∆X_{c}$) and fitting uncertainties ($\sigma_{X_{c}}$) for neat components, the unfilled reference matrix, and glass-filled composites.
