# Supplementary material for: Filler-Geometry-Dependent Crystallinity, Melt Flow, and Mechanical Response of Glass-Filled PHBV + PBAT + TPS Composites
Source: Polymers (Basel). 2026 Sep 13;18(18):2233. doi: 10.3390/polym18182233 (PMC13611261; doi:10.3390/polym18182233)
Supplement: Supplementary file 1 [file polymers-18-02233-s001.zip › polymers-4519566-supplementary.pdf]

Supplementary Materials

Figures

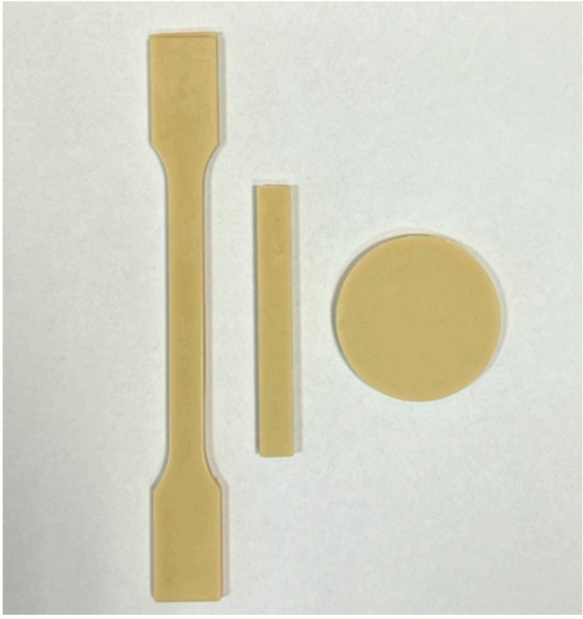

Figure S1: Photo of specimens for investigations of microstructure and properties of the composites

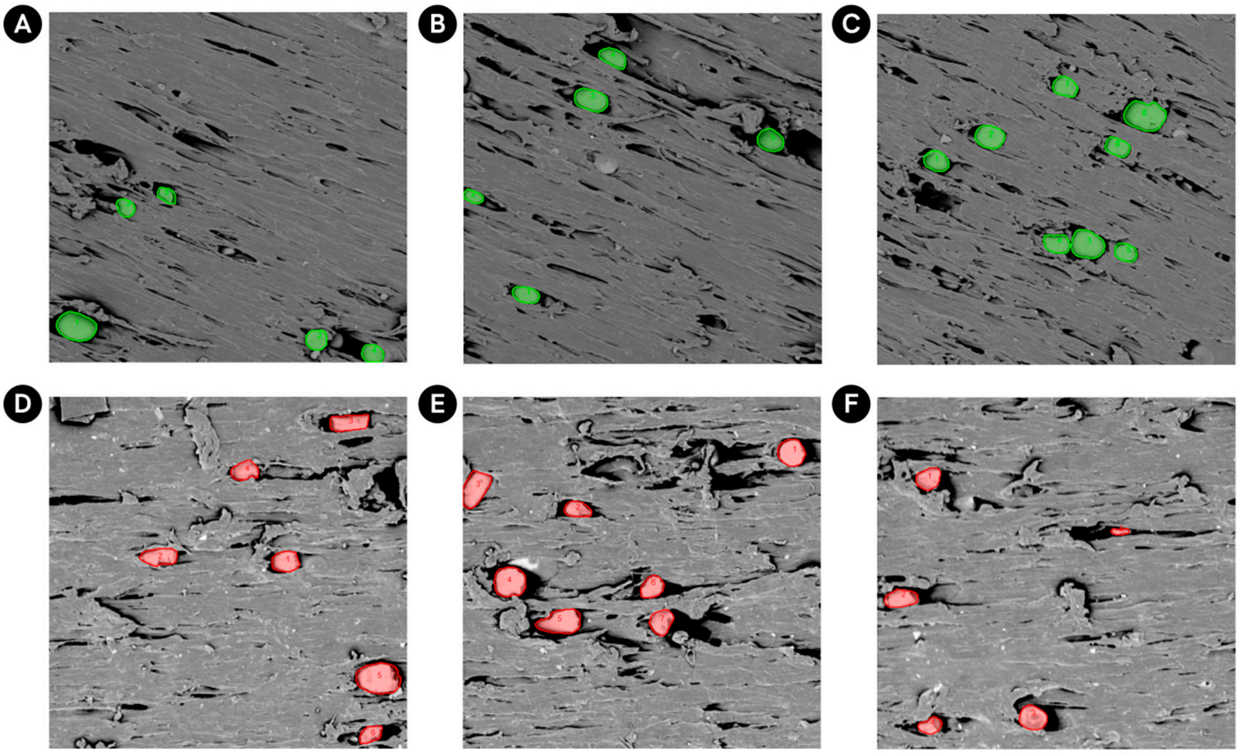

Figure S2: Representative SEM image-processing workflow used for deep-learning-based segmentation. The figure shows examples of raw SEM micrographs, corresponding manual annotations, and segmentation overlays used to identify glass fillers in the PHBV+PBAT+TPS matrix. Panels **(A–C)** present SEM images of glass spheres, with the spheres highlighted in green, whereas panels **(D–F)** show glass fibers, highlighted in red. These data illustrate the procedure applied to extract quantitative stereological parameters, including filler spacing, nearest-neighbour index R, and polymer–glass interfacial area

Tables

Table S1. Manual versus automatic size measurements on the independent validation images. Instances were matched one-to-one at a mask IoU ≥ 0.50. Bias denotes automatic − manual and MAD denotes mean absolute deviation

Detector	Matched pairs	Mean IoU	Descriptor	Manual [µm]	Automatic [µm]	Bias [µm]	MAD [µm]	r
Glass fibers	19	0.84	Equivalent diameter	10.56 ± 2.33	10.49 ± 2.22	−0.07	0.41 (3.9%)	0.973
Glass fibers	19	0.84	Minor-axis diameter	8.39 ± 2.06	7.86 ± 1.66	−0.53	0.77 (9.2%)	0.897
Hollow glass spheres	19	0.78	Equivalent diameter	8.73 ± 2.88	8.95 ± 2.80	+0.22	0.71 (8.1%)	0.950

Table S2. Complete training and inference configuration of the glass-fiber and hollow-glass-sphere detectors

Parameter	Glass-fiber detector	Hollow-glass-sphere detector
Framework	Detectron2 v0.6	Detectron2 v0.6
Architecture	Cascade Mask R-CNN	Mask R-CNN
Backbone	ResNet-50 + FPN	ResNet-50 + FPN
Initialization	cascade_mask_rcnn_R_50_FPN_3x (COCO)	mask_rcnn_R_50_FPN_3x (COCO)
Object classes	1	1
Optimizer	SGD, momentum 0.9, weight decay 1×10^{-4}	SGD, momentum 0.9, weight decay 1×10^{-4}
Base learning rate	0.002	0.002
Iterations	5000	5000
Warm-up	200 iterations, factor 0.001	200 iterations, factor 0.001
Batch size	2 images, gradient accumulation ×4	2 images, gradient accumulation ×4
RoI batch per image	256, positive fraction 0.25	256, positive fraction 0.25
Input size	shortest edge 1024 px, longest edge 2048 px	shortest edge 1024 px, longest edge 2048 px
Augmentation	resize, horizontal flip, brightness/contrast/saturation	resize, horizontal flip, brightness/contrast/saturation
Precision	mixed precision, CUDA GPU	mixed precision, CUDA GPU
Test NMS threshold	0.30	0.30
Maximum detections/image	500	500
Score threshold – quantitative analysis	0.15	0.15
Score threshold – COCO metrics	0.05	0.05

Rotational augmentation was not applied because it would alter the orientation characteristics of the anisotropic glass fibers. The score threshold of 0.15 used for quantitative inference was set explicitly during analysis and overrides the default value of 0.20 stored in the saved configuration files.

Table S3. Multi-peak Voigt deconvolution goodness-of-fit statistics (R^2, χ_v^2), flow-induced planar texture indices (A_{020}/A_{110}), and sensitivity comparison between uncorrected (X_c^{raw}) and ICDD-corrected (X_c^{corr}) XRD crystallinity indices alongside discrepancies between those values (ΔX_c) and fitting uncertainties (σ_{X_c}) for neat components, the unfilled reference matrix, and glass-filled composites

Sample	R^2	WSSR/DoF χ_v^2	A_{020}/A_{110}	X_c^{raw} [%]	X_c^{corr} [%]	ΔX_c [pp]	σ_{X_c} [pp]
**Neat PHBV**	0.9916	231.0	1.50	92.3	77.4	−14.9	±1.1
**Neat PBAT**	0.9983	37.8	—	37.5	37.5	0.0	±0.9
**Neat TPS**	0.9934	242.5	—	11.3	11.3	0.0	±0.8
**Neat PHBV+PBAT+TPS**	0.9961	100.9	2.41	62.9	59.1	−3.8	±1.2
**GF-2**	0.9968	81.6	2.07	64.7	58.4	−6.3	±1.1
**GF-4**	0.9954	82.7	2.98	67.4	58.6	−8.8	±1.2
**GF-10**	0.9961	61.1	1.47	50.9	47.3	−3.6	±1.2
**GF-15**	0.9962	42.5	1.83	49.5	47.4	−2.1	±1.2
**GS-2**	0.9963	70.8	2.08	69.9	60.5	−9.4	±1.1
**GS-4**	0.9973	46.9	2.15	61.9	56.7	−5.2	±1.1
**GS-10**	0.9967	50.3	2.21	55.9	50.9	−5.0	±1.2
**GS-15**	0.9967	42.6	1.12	47.5	47.3	−0.2	±1.2
